# Polydopamine nanoparticles restore cognition via targeted dopamine delivery and septo-hippocampal cholinergic activation

**DOI:** 10.7150/thno.121735

**Published:** 2026-01-01

**Authors:** Pan-Miao Liu, Yu-Ge Wang, Ting-Ting Zhu, Ji-Chun Zhang, Han-Wen Gu, Hui-Juan Li, Wei-Tong Pan, Yan-Bo Zhou, Kui-Cheng Zhu, Kenji Hashimoto, Jian-Jun Yang

**Affiliations:** 1 Department of Anesthesiology, Pain and Perioperative Medicine, The First Affiliated Hospital of Zhengzhou University, Zhengzhou 450052, China.; 2 Laboratory Animal Center of Zhengzhou University, Zhengzhou 450052, China.; 3 Institute of Brain Science and Brain-inspired Research, Shandong First Medical University & Shandong Academy of Medical Sciences, Jinan 250117, China.; 4 Department of Physiology, School of Medicine, Jinan University, Guangzhou 510632, China.; 5 Chiba University Center for Forensic Mental Health, Chiba 260-8670, Japan.; 6 Department of Anesthesiology and Perioperative Medicine, The First Affiliated Hospital of Nanjing Medical University, Nanjing 210029, China.

**Keywords:** cognitive dysfunction, dopamine supplementation, acetylcholine, nanoparticles, hippocampus

## Abstract

Altered dopamine (DA) neurotransmission in key brain circuits underlies cognitive deficits across psychiatric and neurological disorders by disrupting working memory, attention, and executive function. Here, we introduce a novel, carrier-free nanotherapeutic approach using polydopamine nanoparticles (PDA NPs)—synthesized via oxidative self-polymerization of DA hydrochloride—for targeted DA supplementation and cognitive rescue. Uniform, spherical PDA NPs (~250 nm) exhibit excellent biocompatibility and cross the blood-brain barrier via endocytosis. In acidic environments, they degrade to release DA, which is internalized by endothelial and neuronal cells and subsequently converted into downstream catecholamines. In a mouse model of lipopolysaccharide-induced cognitive impairment, PDA NP treatment fully restored performance in Y-maze and novel-object recognition tests. Biochemical analyses showed that short-term administration elevated hippocampal DA, norepinephrine, and tyrosine, while prolonged treatment markedly increased acetylcholine levels. This long-term cholinergic enhancement was mediated by activation of septo-hippocampal projections via DA D2 receptor signaling in the medial septal nucleus. Together, these results establish PDA NPs as an effective, carrier-free platform for targeted DA delivery that not only replenishes catecholamines but also engages cholinergic circuits to ameliorate cognitive impairments.

## Introduction

Psychiatric and neurological disorders that affect cognitive function have complex origins and challenging treatments. Disorders such as depression, schizophrenia, attention-deficit hyperactivity disorder (ADHD), restless legs syndrome, dystonia, Parkinson's disease, and various forms of dementia (including Alzheimer's) are often associated with a deficiency of catecholamines like dopamine (DA) in the central nervous system (CNS) 1-3. Despite extensive efforts to develop catecholamine-based drugs, current therapies face several limitations. First, the blood-brain barrier (BBB) prevents hydrophilic, macromolecular, and highly polar drugs from entering the CNS. Second, systemic enzymes can alter the metabolism of many drugs—especially lipophilic, small molecules and polar compounds—thereby reducing their effectiveness. Third, high levels of drugs that cross the BBB may damage delicate neurons, sometimes causing irreversible harm 4-7. These challenges highlight the urgent need for safe and effective exogenous catecholamine supplements.

Polydopamine (PDA) nanoparticles (PDA NPs) are produced through the oxidative self-polymerization of DA and have gained popularity in various biomedical applications, including drug delivery, biological detection, imaging, disease diagnosis, and antimicrobial treatments 8-12. In the context of drug delivery, PDA NPs are particularly valuable because of their simple synthesis, excellent biocompatibility and stability, versatile modification options, and remarkable ability to cross the BBB 13-15. Moreover, they have shown promise as standalone therapeutic agents for conditions such as periodontitis, ischemic stroke, colitis, and inflammation-related depression, primarily due to their abundant phenol hydroxyl groups that provide effective free radical scavenging 16-19.

PDA forms as a supramolecular polymer through the oxidative self-polymerization of DA, incorporating both covalent and non-covalent bonds 20. Studies indicate that about 14.2% of DA remains unpolymerized within this structure 21. This observation suggests that under acidic conditions, PDA could dissociate to release catecholamines. Importantly, PDA NPs can cross the BBB via endocytosis. Once in the brain, they may accumulate in acidified endosomes or lysosomes, where the unpolymerized DA is released 11,22. Thus, these carrier-free PDA NPs not only deliver their payload but also self-degrade in the process 16-19. However, research on using PDA NPs specifically for targeted DA delivery in treating psychiatric and neurological disorders is still limited.

In this study, we explored the therapeutic potential of PDA NPs for enhancing cognitive function in mice. We synthesized PDA NPs with diameters of 200-300 nm through DA oxidation and self-polymerization. Their biological activity and ability to cross the BBB were evaluated in both *in vitro* and *in vivo* experiments. We also conducted a metabolomics analysis to assess catecholamine production after injection, and behavioral tests to examine the effects of PDA NPs on cognitive deficits in lipopolysaccharide (LPS)-treated mice. Finally, we investigated the neural circuits and cellular mechanisms underlying both the short- and long-term cognitive benefits of PDA NP treatment.

## Methods

### Chemical and materials

Dopamine (DA) hydrochloride, ammonium hydroxide (NH_4_OH), ethanol, N,N-dimethylformamide (DMF), cetyltrimethylammoniumchloride (CTAC), triethanolamine (TEA), tetraethyl orthosilicate (TEOS) and Cyanine5.5 (Cy5.5) NHS ester were obtained from Aladdin (Shanghai, China). SCH23390 hydrochloride and Lipopolysaccharide (LPS) were purchased from Sigma-Aldrich (St Louis, MO, USA). Raclopride was purchased from MedChemExpress company (NJ, USA). BRL-3A cells, PC-12 cells, HBZY-1cells, RAW264.7 cells, Minimum Essential Medium (MEM) and fetal bovine serum (FBS) were achieved from Procell Life Science & Technology. Deionized water (18.0 MΩ cm, Milli-Q gradient system, Millipore) was used in all experiments. All chemical reagents were used without further purification. All reagents were of analytical grade and used as received.

### Preparation of PDA nanoparticles (NPs)

NH₄OH (0.6 mL), absolute ethanol (32 mL), and deionized water (72 mL) were mixed and gently stirred for 30 min at room temperature. 10 mL of dopamine (DA) hydrochloride (400 mg) solution introduced into the mixture to initiate polymerization reaction, which proceeded for 18 h. The resulting polydopamine nanoparticles (PDA NPs) were subsequently purified via dialysis against deionized water using 8,000 Da molecular weight cut-off dialysis membranes for 5 days.

### Preparation of PDA@Cy_5.5_@MSN

First, 8 mL of a 25% CTAC aqueous solution and 0.06 g TEA were added to 20 mL of water. The mixture was heated at 95 ℃ and stirred for 1 h. Then, 1.5 mL TEOS was added dropwise at a rate of 0.15 mL/min, and stirring continued for another 1 hour. The resulting product was centrifuged at 10,000 rpm for 30 min, and the supernatant was discarded. The residue was dried and subjected to Soxhlet extraction with hydrochloric acid ethanol solution for 48 hours. Next, 0.07 g of MSN was dissolved in 6 mL of DMF and dispersed using ultrasound. To this, 50 µL of Cy_5.5_-NHS/DMF stock solution (2 mg/mL DMF) was added. The mixture was kept in the dark, stirred at 600 rpm, and reacted for 48 h at room temperature. It was then centrifuged at 10,000 rpm for 30 min. The supernatant was added to a mixed solution containing 12 mL water, 8 mL ethanol, 300 µL DA, and 150 µL of ammonia, and stirred at room temperature for 24 h. Finally, the mixture was centrifuged at 10,000 rpm for 30 min to obtain PDA@Cy_5.5_@MSN.

### Characterization

The morphology of PDA NPs was examined using a field emission scanning electron microscopy (SEM, ZEISS Sigma 300). The zeta potential of both PDA NPs and PDA@Cy_5.5_@MSN was determined using a Zetasizer Nano ZS (Malvern Instruments Ltd, NANO ZS90). The structural details of PDA@Cy_5.5_@MSN were analyzed using Transmission Electron Microscopy (TEM, JEM2100F).

### Cytotoxicity assay

BRL-3A, PC-12, HBZY-1, and RAW264.7 cells were seeded into 96-well plates at 5,000 cells per well and cultured for 24 h in MEM supplemented with 10% FBS. The culture medium was then replaced with fresh medium containing PDA NPs at concentrations of 0, 10, 50, or 100 μg/mL, followed by incubation for 12, 24, or 48 h. After treatment, the medium containing PDA NPs was discarded, and cells were washed twice with fresh medium to prevent potential interference of residual nanoparticles with absorbance detection. Subsequently, 90 μL of fresh medium and 10 μL of CCK-8 reagent (Shanghai, China) were added to each well and incubated for 1 h. The optical density at 450 nm was recorded using a microplate reader (Bio-Rad Laboratories Inc.). Cell viability was calculated as: Cell viability (%) = (CD of the experimental group/CD of the control group) × 100%.

### Animals

All experimental procedures were approved by the Ethics Committee of Zhengzhou University. Two hundred male C57BL/6J mice (8 weeks old) were obtained from the Animal Center of Zhengzhou University (Zhengzhou, China). Only male mice were used to minimize the influence of hormonal fluctuations. Animals were group-housed (five per cage) under controlled conditions (24 ± 1 °C; 12-h light/dark cycle) with ad libitum access to standard chow and water. All procedures complied with the National Institutes of Health Guidelines for the Care and Use of Laboratory Animals.

### LPS-induced cognitive impaired model

LPS (0.5 mg/kg) was dissolved in normal saline and administered intraperitoneally (i.p.). Based on previous reports, a single LPS injection at this dosage is sufficient to induce cognitive impairment in mice 23.

### Behavioral tests

A trained investigator, blinded to the experimental group, conducted the behavioral tests in a sound-isolated room. The tests included the open field test (OFT), Y-maze, and novel object recognition (NOR).

Open field test (OFT): Mice were placed at the center of a white open-field chamber (50 × 50 × 50 cm) in a dimly lit room and allowed to freely explore for 5 min. Locomotor activity was recorded using the SMART 3.0 video tracking system (Panlab/RWD, Shenzhen, China). Data recorded included total distance moved, time spent in the center, and the number of entries into the center.

Y-maze: The Y-maze test assessed spontaneous alternation, which reflects the animals' exploratory behavior and preference for new arms of the maze. The apparatus consisted of three enclosed arms arranged at 120° angles, each measuring 28 cm in length, 6 cm in width, and 18 cm in height. Mice were placed in the center of the maze, and their behavior was tracked for 8 minutes in a quiet, dimly lit room (30 lux). A blinded investigator recorded the sequence and number of arm entries. Spontaneous alternation was defined as a mouse visiting all three arms in consecutive choices (ABC, BCA, or CAB), but not repeating any arm immediately (e.g., BAB, CAC). The percentage of spontaneous alternation was calculated as follows: % spontaneous alternation = number of spontaneous alternations/(total arm entries-2) *100.

Novel object recognition (NOR): NOR testing was conducted in the same arena as the OFT to minimize the need for extended habituation. The session began with a 10-minute training phase, during which the mice explored two identical objects. After a 24-hour inter-trial interval (ITI) in their home cage, mice were exposed to a retention test for 5 minutes, which involved one familiar object and a novel object. The longer the mice spent exploring the novel object, the stronger the evidence for intact recognition memory. Exploration was defined as sniffing or touching the object with the nose, and the total time spent within 1.5 cm of each object was recorded. The discrimination index was calculated as:

Discrimination index = (novel object investigation time)/(total investigation time for both objects) × 100. All trials were video-recorded and analyzed using the computerized tracking system (Shanghai Softmaze Information Technology Co. Ltd, Shanghai, China)

### *In vivo* imaging

Due to the fluorescence quenching effect of PDA NPs, which impeded direct in vivo imaging, we utilized PDA@Cy_5.5_@MSN as a proxy for Cy_5.5_-labled PDA NPs.This was accomplished by utilizing mesoporous silica nanoparticles to adsorb Cy_5.5_ fluorescent molecules through non-covalent interactions. Subsequently, DA was subjected to oxidative self-polymerization to form a PDA shell around the PDA@Cy_5.5_@MSN. These nanoparticles were then injected i.p. to evaluate the ability of PDA NPs to cross the BBB. Fluorescence intensity from Cy_5.5_ was monitored at 2 and 24 h post-injection using an *in vivo* imaging system (IVIS Spectrum, PerkinElmer, USA), to track the entry of nanoparticles into the brain.

### Immunohistochemistry

Mice were deeply anesthetized using isoflurane and subsequently perfused slowly with phosphate-buffered saline (PBS) followed by 4% paraformaldehyde (PFA). Their brains were then carefully removed and post-fixed in 4% PFA overnight. For dehydration, the brains were immersed in 30% sucrose solutions in PBS, and then coronally sectioned into 25 µm-thick slices using a cryostat (CM1950, Leica). To block these sections, a solution of 5% normal goat serum and 0.3% Triton X-100 in PBS was applied for 1 hour at room temperature. This was followed by overnight incubation at 4 °C with primary antibody: rabbit anti-choline acetyltransferase (1:500, Abcam, USA, ab178850). The sections were washed three times with PBS and incubated with goat anti-rabbit Cy_3_-conjugated secondary antibody (1:400, Jackson ImmunoResearch, USA, 111-165-003) for 2 h at room temperature. For nuclear staining and localization, the slices were immersed in 10 μg/mL 4',6-diamidino-2-phenyl-indole (DAPI) (C0065, Solarbio) for 5 min. Fluorescence microscopy images were then acquired using a confocal scanning microscope (A1 MP+, Nikon).

### Hemolysis assay

Red blood cells were isolated from serum and diluted to a 2% concentration with PBS. We prepared three mixtures: 1 mL of the diluted red blood cells with PBS (1 mL) as negative control, 1 mL of the diluted cells with 1 mL of 0.2% Triton X-100 as positive control, and 1 mL of the diluted cells with 1 mL of 200 μg/mL PDA NPs as the experimental sample. Each mixture was incubated at 37 ℃ for 4 h and then centrifuged at 1000 rpm for 15 min. The absorbance of the supernatants at 545 nm was subsequently measured using a Bio-Rad microplate reader.

### Hematoxylin-eosin (H&E) staining

The major organs (heart, liver, spleen, lung, kidney) were fixed with 4% PFA, and then embedded in paraffin. These samples were sectioned into 4 μm thick slices using a microtome (RM2016, Shanghai Leica Instrument), followed a graded alcohol dehydration process. Hematoxylin and eosin (H&E) staining was then performed on these sections, and the pathological changes in the major organs were examined using a light microscope at 200× magnification.

### Surgery

Animals were anesthetized by i.p. injection of tribromoethanol (200 mg/kg) and then secured in a stereotaxic frame (Stoelting Instruments, USA) in a flat skull position with the incisor bar set to -3.3 mm relative to the interaural line. A midline incision was made, and the skin and underlying periosteum were retracted. Stainless steel guide cannulae (26 gauge) were implanted 1 mm above the target sites using the following stereotaxic coordinates for the medial septum: anterior-posterior (AP) 0.95 mm from bregma, lateral (L) ± 0.2 mm from the midline, and ventral (V) -4.5 mm relative to the dura. The guide cannulae were secured to the skull with jeweler's screws and dental cement. Prior to microinjections, stainless steel stylets (26 gauge) of varying lengths were inserted into the guide cannulae to maintain patency. After surgery, animals were housed individually, and incision sites were disinfected periodically with iodophor-soaked cotton swabs. Three weeks later, all animals survived and successfully completed the subsequent microinjection procedures.

### Microinjection

A 26-gauge injection cannula—1 mm longer than the guide cannulae—was lowered so that it extended 1 mm beyond the tip of the guide cannulae, targeting the center of the intended site. Intra-MS injections were performed with a volume of 0.2 μL per injection. Each microinjection was delivered over 60 s, after which the injection cannula was left in place for an additional 120 s to allow for proper diffusion of the drug.

To evaluate the effects of post-PDA NP administration, intra-MS injections of DA receptor antagonists were performed. Raclopride hydrochloride (a D2 receptor antagonist, 1 mg/mL) and SCH23390 hydrochloride (a D1 receptor antagonist, 1 mg/mL) were used. Animals were divided into four groups and initially administered i.p. injections of saline or PDA NPs. On days 1, 3, 5, and 7 after the first PDA NP administration, the animals received intra-MS injections as follows: Group 1 and Group 2 received PBS, Group 3 received raclopride, and Group 4 received SCH23390 (0.2 μL/mouse/day).

### Microdialysis

Microdialysis and ultra-performance liquid chromatography-mass spectrometry (UPLC-MS/MS) were performed as described. To measure extracellular DA, norepinephrine (NE), acetylcholine (ACh), and tyrosine in the hippocampus, a microdialysis probe was inserted into the dorsal hippocampus (AP -2.0 mm, ML -1.3 mm, DV -2.3 mm) via a guide cannula under anesthesia. Microdialysis commenced by connecting the probe to a microinjection pump system (CMA, Stockholm, Sweden). The probe was continuously perfused with non-buffered artificial cerebrospinal fluid (147 mM NaCl, 4 mM KCl, 1.2 mM CaCl₂, 1.2 mM MgCl₂) at a rate of 1 μL/min. For both PBS- and PDA NPs-treated mice, the initial 60 min of perfusate were discarded to ensure system equilibrium, after which samples were collected for at least one additional hour. All collected microdialysis samples were immediately frozen at -80 °C and stored until UPLC-MS/MS analysis (Waters Acuity, Waters, USA).

### DPPH radical scavenging assay

A 0.1 mM DPPH solution was prepared in 95% ethanol. Two milliliters of this solution were then combined with PDA NPs at a final concentration of 0.2 mg/mL. The mixture was incubated in the dark, and the decrease in absorbance at 517 nm was measured at different time points (10, 30, 60, 120, and 240 min) to assess radical scavenging activity. The DPPH scavenging rate was calculated using the following equation: I=[1-(A_i_-A_j_)/A_c_] × 100%, where A_c_ is the absorbance of the DPPH solution alone, *Ai* is the absorbance of the nanoparticle-DPPH mixture at 0 min, and *Aj* is the absorbance of the sample mixed with 95% ethanol.

### Intracellular ROS scavenging assay

To assess the ROS-scavenging capacity of PDA NPs *in vitro*, PC12 cells were co-incubated with PDA NPs. PC12 cells were initially plated in 12-well plates at a density of 10,000 cells per well and incubated for 12 hours. After medium removal, PDA NPs (100 μg/mL) were added in MEM supplemented with 10% FBS and incubated for 6 h. Cells were then rinsed with PBS, followed by incubating with LPS (1.0 mg/mL) for 30 min. After LPS treatment, cells were washed again with PBS and subsequently incubated with DHE at 37 °C for 30 min in the dark. Untreated cells served as the control group. Fluorescence images were captured using an Olympus microscope imaging system.

### UPLC-MS/MS analysis

Chromatographic conditions were set using a Waters Acuity Ultra High-Performance Liquid Chromatograph (Waters) equipped with an ACQUITY BEH Amide column (2.1 mm × 100 mm, 1.7 μm). The mobile phases comprised acetonitrile (B) and 0.2% formic acid in water (A). Gradient elution conditions were as follows: 0-1.2 min at 22% A; 1.2-3.2 min with a linear increase from 22% to 60% A; 3.2-4.0 min at 60% A; and 4.0-5.0 min at 22% A. The column temperature was maintained at 35 ℃, with a flow rate of 0.35 mL/min and an injection volume of 10 μL.

For mass spectrometric detection, an electrospray ionization (ESI) source was used in both positive and negative ion scanning modes. The ion source spray voltage ranged from -5.5 kV to 5.5 kV. The gas parameters were set as follows: curtain gas at 241.3 kPa, collision (impact) gas at 48.3 kPa, atomizing gas at 413.7 kPa, and auxiliary gas at 413.7 kPa. The ion source temperature was 500 ℃, and the analysis time for each sample was 3.5 min. In multi-reaction monitoring (MRM) mode, instrument parameters and the monitored ion pairs are detailed in the accompanying table (**Table 1**).

### Fibre photometry

Sixteen days before PDA NP or PBS injections, rAAV-hSyn-ACh3.0 was stereotactically injected into the right dorsal hippocampus (AP -2.0 mm, ML -1.3 mm, DV -1.6 mm) to monitor ACh release. An optical fiber was then implanted into the right dorsal hippocampus for fluorescence monitoring using a multichannel fibre photometry system (Inper LLC, Hangzhou, Zhejiang, China). After selecting the recording channel, the system was set with a sampling rate of 30 Hz, an exposure time of 15 ms, a gain of 0, 470 nm LED light power at 45%, and 410 nm LED light power at 15%, resulting in an approximate light output of 10-15 mW. Signals were recorded for 10 min while the mice were freely moving. A baseline recording was taken on D1 (before the first injection of PDA NPs or PBS) and subsequent recordings were made on D4, D7, and D9. Photometry data were exported to Excel for analysis using Origin and GraphPad Prism software. Using the 410 nm channel as a baseline reference (environmental noise), the F_470_/F_410_ ratio (denoted as F_i_) was calculated to normalize the raw signals. Finally, the fluorescence change (∆F/F) was derived using the formula (F_i_ - F_B_) / F_B_, where Fi includes the signals from D4, D7, and D9, and FB is the baseline signal from D1. These values were then presented as heatmaps or average plots.

### Chemogenetics

Animals were anesthetized i.p. with tribromoethanol (200 mg/kg) and secured in a stereotaxic frame (Stoelting Instruments, USA) in a flat skull position with the incisor bar set at -3.3 mm relative to the interaural line. For injections into specific brain regions, the following bregma coordinates were used: right dorsal hippocampus (AP -2.0 mm, ML -1.3 mm, DV -1.6 mm) and medial septum (AP 0.95 mm, ML ± 0.2 mm, DV -4.5 mm). Glass cannulas filled with virus solution were lowered to the specified depth from the dura, and 0.5-1.5 µL of virus solution was injected using a microinjection pump (Harvard Apparatus) at a flow rate of 0.1-0.25 µL/min. The adeno-associated viruses (AAVs) used for stereotactic injections were purchased from BrainVTA (China) and included: rAAV-ChAT-CRE-WPRE-hGH polyA, AAV2/R; rAAV2-EF1α-DIO-hM4D(Gi)-mCherry-WPRE, AAV2/R; and rAAV2-EF1α-DIO-mCherry-WPRE, AAV2/R. All mice received an injection of clozapine-*N*-oxide (CNO) (1.0 mg/kg, i.p., MedChemExpress, HY-17366) 30 min before behavioral testing.

### Statistical analysis

Statistical analysis and plot generation were performed using GraphPad Prism software (version 8). Data are expressed as the mean ± standard error of the mean (SEM). To assess group differences, one-way analysis of variance (ANOVA) was conducted, followed by Bonferroni's post hoc test for multiple comparisons. Statistical significance was defined as *P < 0.05, **P < 0.01, ***P < 0.001.

## Results

### Preparation and characterization of PDA NPs

**Figure 1:**
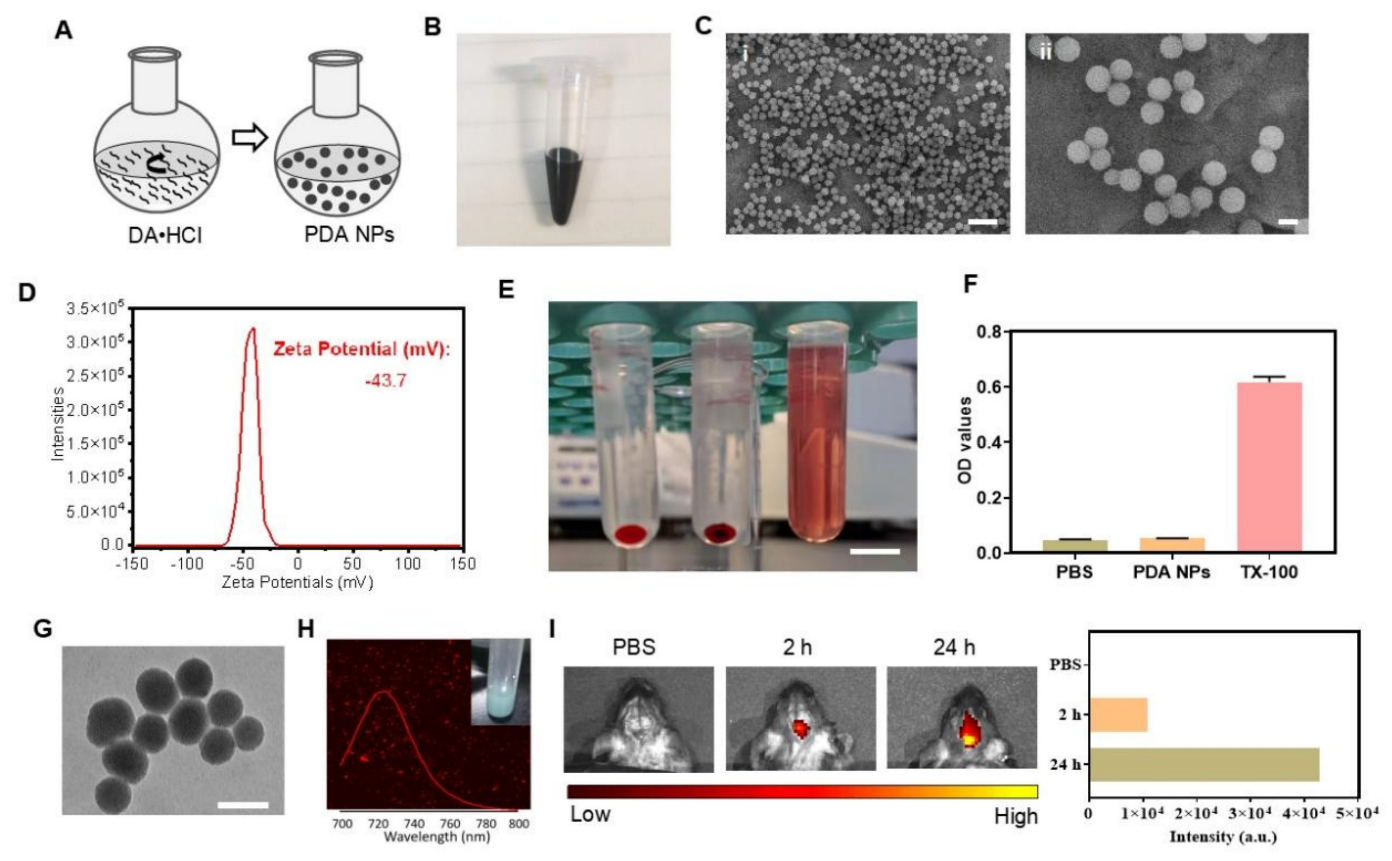
** Preparation and BBB permeability of PDA NPs.** (A): Schematic diagram illustrating the synthesis process of PDA NPs. (B): Photograph showing the suspension of PDA NPs. (C): SEM images of PDA NPs (scale bar: i: 1 μm; ii: 200 nm). (D): Zeta potential analysis of PDA NPs. (E): Representative image of the hemolytic reaction of red blood cells in different solutions (scale bar: 1 cm). (F): Bar graph quantifying the hemolysis observed in various solutions (n = 3 per group). PBS, PDA-NPs (200 μg/mL), TX-100 (0.2%). (G): TEM image of PDA@Cy_5.5_@MSN. Scale bar: 200 nm. (H): Fluorescence spectrum and photograph of PDA@Cy_5.5_@MSN suspension. (I): The fluorescent intensity and the corresponding bar graphs of PDA@Cy_5.5_@MSN in the brain of mice.

We synthesized PDA NPs via the oxidative self-polymerization of DA hydrochloride (DA·HCl), as illustrated in **Figure 1A** 24. A 30-h reaction yielded black and brown PDA NPs (**Figure 1B**). Scanning electron microscopy (SEM) confirmed that the particles were uniformly spherical, with an average diameter of approximately 250 nm (**Figure 1C**). Zeta potential measurements revealed a surface charge of -43.7 mV (**Figure 1D**). In a hemolysis assay, even at 200 μg/mL, PDA NPs did not cause significant hemolysis after 4 h—similar to the PBS negative control (**Figure 1E-F**). Positive control TX-100 caused significant hemolysis (**Figure 1E-F**). Together, these results confirm that PDA NPs were successfully synthesized with excellent biocompatibility.

### BBB permeability of PDA NPs

To assess whether PDA NPs can cross the BBB, we tracked surrogate nanoparticles. Because PDA inherently quenches fluorescence, we used Cy_5.5_-labeled mesoporous silica nanoparticles (MSN) (**Figure S1**) coated with a PDA shell (PDA @Cy_5.5_@MSN) for tracking (**Figure S2A**). Transmission electron microscopy (TEM) showed that these particles were spherical with an average size of 224 nm (**Figure 1G**) and had a zeta potential of -38.2 mV (**Figure S2B**), similar to the PDA NPs (**Figure 1C-D**). Fluorescence imaging confirmed strong red emission at 720 nm (**Figure 1H**). After i.p. injection in mice, *in vivo* imaging revealed obvious fluorescence in the brain at 2 and 24 h post-injection, indicating efficient BBB penetration (**Figure 1I**). Further, mice injected with 10 mg/kg PDA NPs showed brownish-black NPs in 1-μm-thick brain sections three days later, unlike the PBS control (**Figure S3**). PDA NPs were also observed in the liver, spleen, and kidney (**Figure S3**). In cell co-culture experiments, PDA@Cy_5.5_@MSN adhered to cell membranes—likely because of their negative charge—and some particles were internalized within 1 h, suggesting endocytosis (**Figure S4**). Together, these findings demonstrate that PDA NPs can cross the BBB and enter the brain.

### Release of small granules and DA in the acid medium by PDA NPs

**Figure 2:**
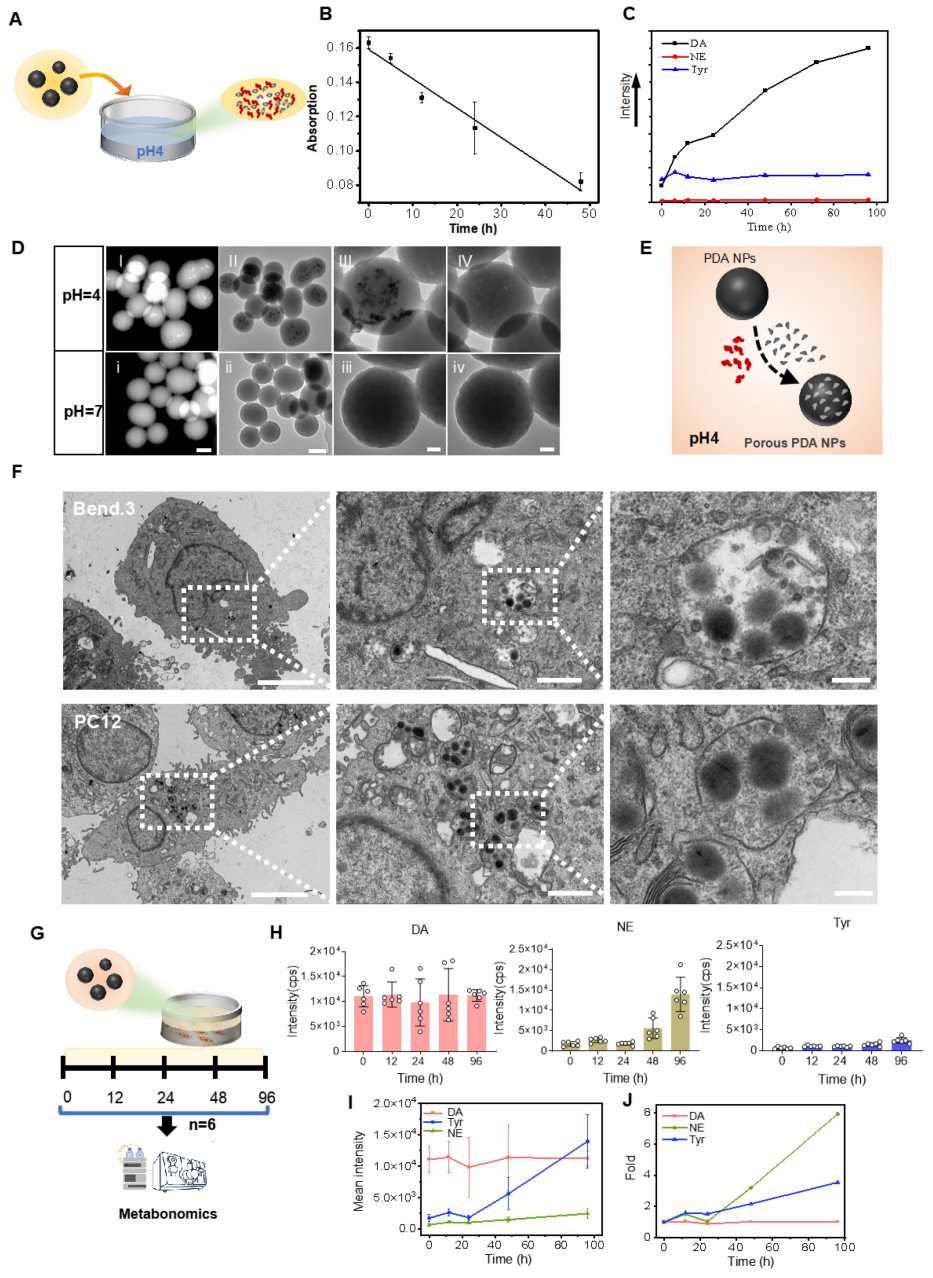
** PDA NPs released small granules and DA in the acid medium and penetrated the cells and subsequently released DA and NE.** (A): Schematic illustrating PDA NPs immersed in an acidic solution. (B): Graph depicting the correlation between the absorption rate at 350 nm and the duration of agitation in the acidic solution. (C): Curves representing the relative levels of DA, NE, and tyrosine from PDA NPs in an acidic solution over time (0, 6, 12, 24, 48, 72, and 96 h). (D): HADDF images (i and I) and TEM images (ii-iv and II-IV) of PDA NPs after 96 h in solutions at pH 4 and pH 7, respectively (scale bar: 50 nm). (E): Schematic representation hypothesizing the degradation process of PDA NPs in an acidic solution. (F): TEM images of PDA NPs after 12 h of co-culture with bend.3 and PC12 cells. The left two images have a scale bar of 5 μm, the middle two images a scale bar of 1 μm, and the right two images a scale bar of 200 nm. Intracellular PDA NPs are highlighted within rectangular dotted boxes. (G): Schematic illustrating the co-culture of PDA NPs with bend.3 cells for metabolomic analysis. (H): Bar graph showing the relative levels of DA, NE, and tyrosine. (I): Curves displaying the relative levels of DA, NE, and tyrosine measured at 0, 12, 24, 48, and 96 h. (J): Fold changes in DA, NE, and tyrosine over time. The sample size for panels E and F is n = 6.

To test DA further decomposition and release from PDA under an acidic environment 25, PDA NPs were exposed to a dilute hydrochloric acid solution (pH 4) and the catecholamine content in the supernatant was monitored (**Figure 2A**). Ultraviolet-visible spectra showed a gradual decrease in absorption between 200 and 550 nm during continuous agitation in acid (**Figure S5**), and a negative linear correlation between absorption at 350 nm and agitation time confirmed PDA NP degradation in acid (**Figure 2B**).

HPLC analysis revealed that, similar to melanin degradation into naturally occurring molecules such as DA and tyrosine 26, DA and tyrosine were detectable in the supernatant—while NE was nearly absent. Notably, DA levels increased about ninefold after 96 h, whereas tyrosine levels remained stable (**Figure S6** and** Figure 2C**). TEM and high-angle annular dark-field (HAADF) imaging after 96 h of degradation in acid showed the formation of smaller granules within the PDA NPs (**Figure 2D (I-III)** and** (i-iii)**). Under high-resolution TEM, these granules appeared to detach and leave numerous small pores, although the overall spherical structure remained intact (**Figure 2D (IV)**,** (iv)** and **Figure S7**). These changes were observed only at pH 4, not at pH 7 (**Figure 2H (i-iv)**). **Figure 2E** illustrates this process. In summary, in acidic conditions, PDA NPs release DA and form smaller granules while preserving their spherical integrity.

### Cellular uptake and release of DA and NE by PDA NPs

NPs, including PDA NPs, are believed to enter cells via endocytosis—a mechanism that likely also facilitates their crossing of the BBB 27, 28. Once inside cerebral vascular endothelial cells, PDA NPs encounter endosomes and lysosomes where the pH drops to approximately 5.0-5.5 (**Figure S8A**), promoting PDA degradation. The released DA can then be converted into other catecholamines by cellular enzymes, thereby affecting brain function. To explore this, PDA NPs were co-cultured with bend.3 endothelial cells and PC12 neuronal cells. After 12 h, most PDA NPs adhered to the surface of bend.3 cells, likely due to their strong negative charge, while some particles were internalized (**Figure S8B**). TEM imaging confirmed entry into both cell types (**Figure 2F**).

Neurotransmitter-related metabolomics of the culture supernatant analyzed by HPLC showed sustained high levels of DA—about ten times that of tyrosine—and a notable increase in NE over time (**Figure 2G**). By 96 h, NE levels had increased approximately eightfold and exceeded DA levels, while DA remained stable (**Figure 2H-J** and **Figure S9**). This rise in NE is likely due to the hydroxylation of DA by intravesicular DA β-hydroxylase or its direct synthesis by endothelial cells 29. These results demonstrate that PDA NPs are internalized by cells, releasing DA and tyrosine, and promoting conversion to NE within the cellular environment.

### Effects of PDA NPs on cognitive deficits in LPS-treated mice

To evaluate the therapeutic effect of PDA NPs, LPS-treated mice received i.p. injections of PDA NPs at 5, 10, or 20 mg/kg (**Figure S10A**). Behavioral tests—the OFT, Y-maze, and NOR—showed that locomotor activity remained unchanged (**Figure S10B**). In the Y-maze, mice treated with 5 and 20 mg/kg showed significant improvements in spontaneous alternation compared to LPS-treated controls, whereas the 10 mg/kg merely showed an improvement trend (**Figure S10C**). Similarly, in the NOR test, recognition ratios were significantly higher in the 5 and 20 mg/kg groups compared to the LPS + saline groups (**Figure S10D**), suggesting that PDA NPs at 5 mg/kg can mitigate LPS-induced cognitive deficits.

**Figure 3:**
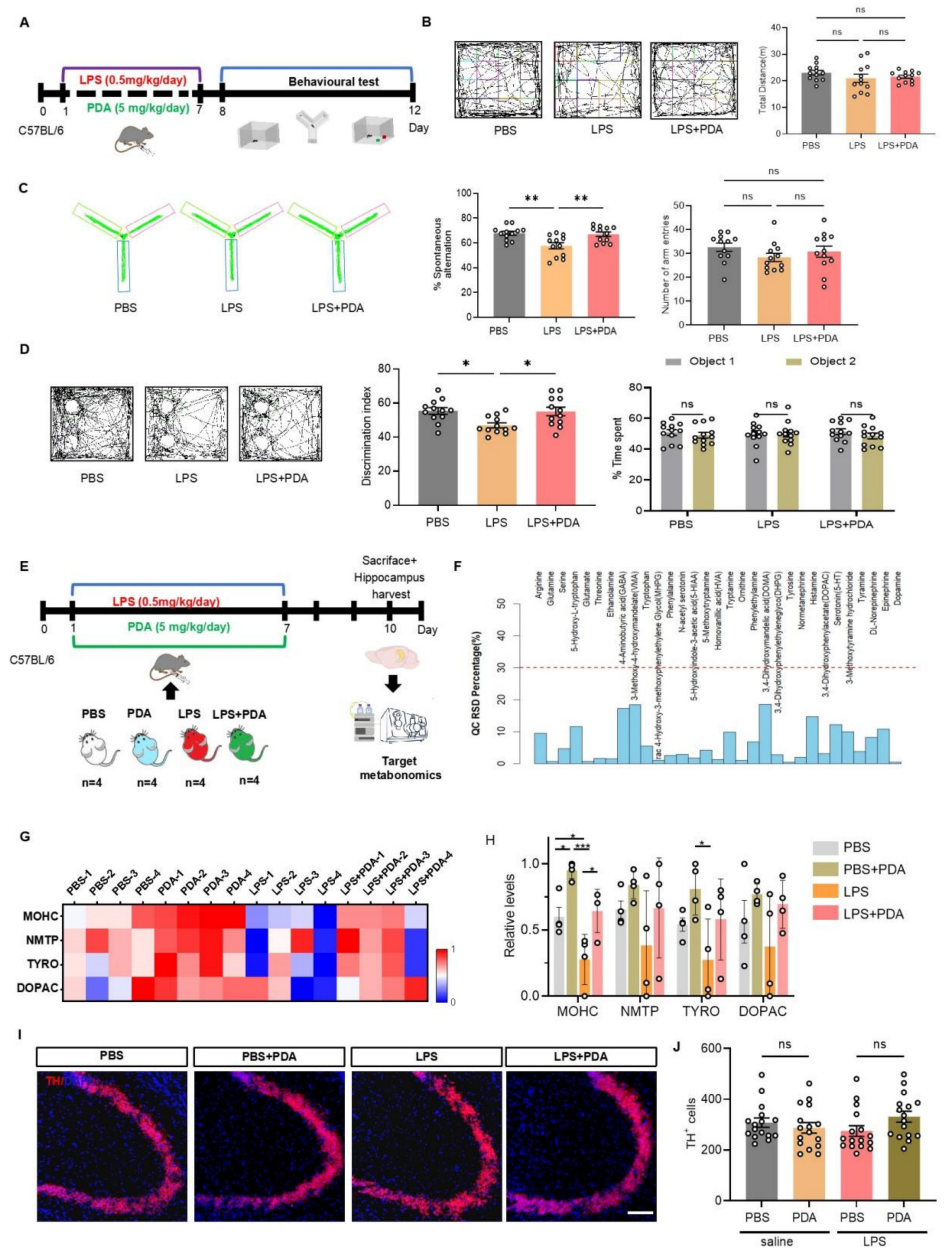
** PDA NPs alleviate cognitive deficits in LPS-treated mice by elevating catecholamine neurotransmitter levels.** (A): Schematic illustrating the experimental design. Mice were i.p. injected with LPS (0.5 mg/kg/day) for seven consecutive days (D1-D7) to induce cognitive deficits. Concurrently, PDA NPs (5 mg/kg/day) were administered for seven consecutive days (D1-D7). Cognitive behaviors were then evaluated using the open field test (OFT), Y-maze, and novel object recognition (NOR) test. (B): Representative activity tracking during the OFT with corresponding bar graphs (with data dots) indicating the total distance traveled during the 5-min test. (C): Representative activity tracking in the Y-maze, along with bar graphs (with data dots) depicting spontaneous alternation performance. (D): Representative activity tracking in the NOR test and corresponding bar graphs (with data dots) displaying the novel object recognition ratio. (E): Schematic outlining the experimental procedure for targeted metabolomics. Mice were treated with LPS (0.5 mg/kg/day, D1-D7) and PDA NPs (5 mg/kg/day, administered on D1-D7). (F): Graph showing the distribution of relative standard deviations (RSDs) in QC samples, indicating that neurotransmitter measurements with an RSD of less than 30% are stable and reliable. (G): Heatmap illustrating the top four changes in catecholamine-related metabolites in the hippocampus: 3-methoxytyramine hydrochloride (MOHC), normetanephrine (NMTP), tyrosine (TYRO), and 3,4-dihydroxyphenylacetate (DOPAC). (H): Corresponding bar graphs with dots represent these changes on day 10. (I): Representative immunofluorescence images, and (J): Corresponding bar graphs (with data dots) showing the number of tyrosine hydrolase (TH)-positive cells in the hippocampus. *P < 0.05, *P < 0.001; n = 4.

In a separate experiment, mice were treated with either PBS or 5 mg/kg PDA NPs (**Figure 3A**). The OFT confirmed unchanged locomotor activity (**Figure 3B**). The Y-maze revealed that PDA NP treatment restored spontaneous alternation in LPS-treated mice to levels comparable to the PBS group (**Figure 3C**). The NOR test also showed that PDA NPs restored recognition ratios in LPS-treated mice (**Figure 3D**), and the weight of these mice exhibited no significant recovery (**Figure S11**). To determine if these cognitive improvements were related to increased catecholamine levels, targeted metabolomics on hippocampal tissue 3 days after the final injection was performed (**Figure 3E**). Quality control samples confirmed data stability (**Figure 3F**). Of 31 metabolites monitored, 14 were linked to the catecholamine system (**Figure S12**). The top four differential metabolites were 3-methoxytyramine, normetanephrine, tyrosine, and 3,4-dihydroxyphenylacetate (**Figure 3G**). Notably, 3-methoxytyramine—a DA metabolite—decreased in LPS-treated mice but increased dramatically following 5 mg/kg PDA NP treatment, with similar trends observed for normetanephrine, tyrosine, and 3,4-dihydroxyphenylacetate (**Figure 3H**). Quantification of tyrosine hydroxylase (TH)-positive neurons in the hippocampus (**Figure 3I**) revealed no significant differences among groups (**Figure 3J**), suggesting that the elevated catecholamine levels resulted from PDA NP treatment rather than increased endogenous synthesis. Collectively, these findings indicate that PDA NPs improve cognitive deficits in LPS-treated mice by elevating catecholamine levels, providing a promising strategy for exogenous catecholamine supplementation. Together, these findings indicate that PDA NPs can improve cognitive deficits in LPS-treated mice by elevating catecholamine levels, offering a promising alternative for exogenous catecholamine supplementation in the treatment of cognitive impairments.

### Long-term enhancement of hippocampal Ach by PDA NPs in mice

**Figure 4:**
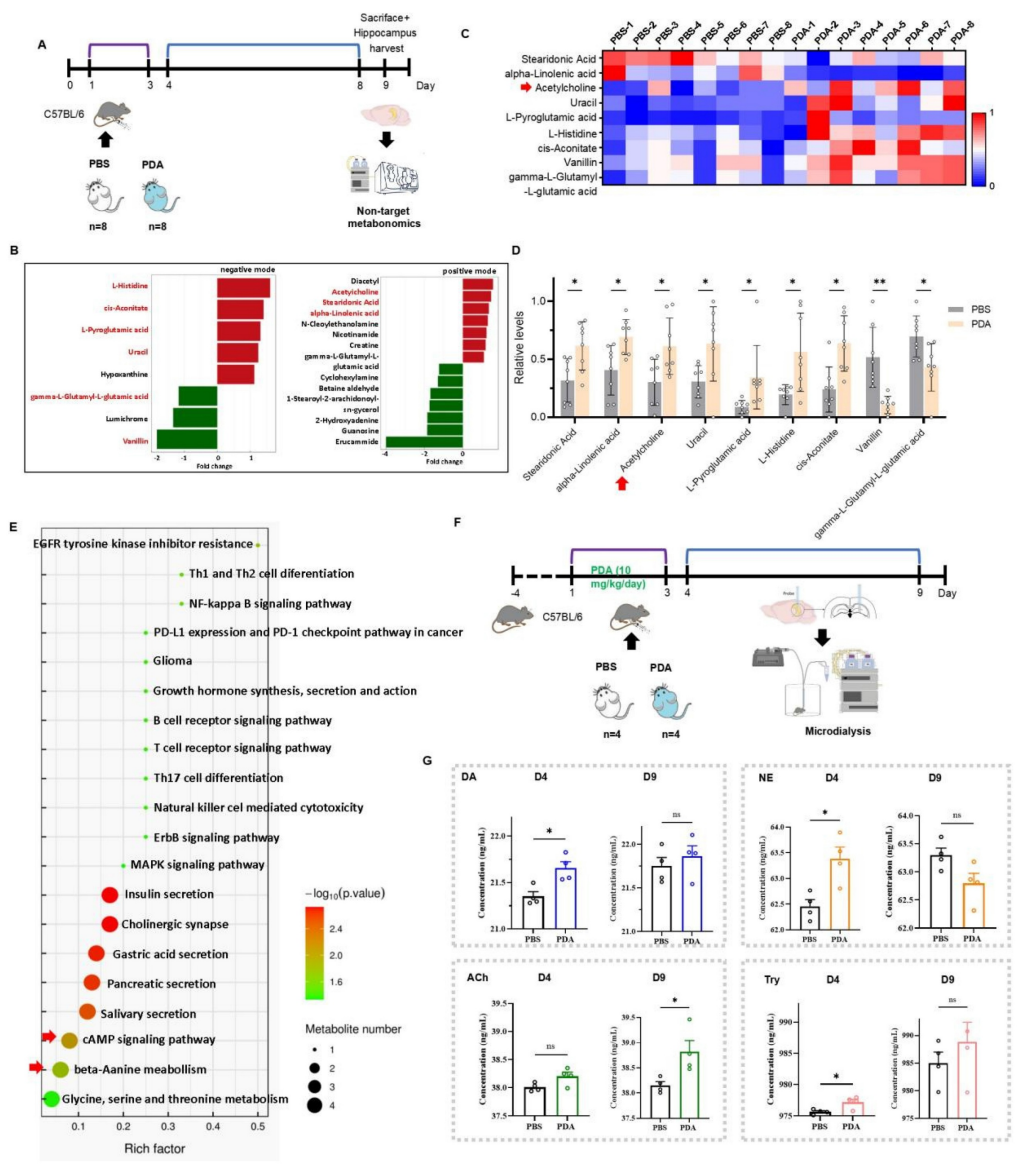
**PDA NPs induce a long-term elevation of ACh in the hippocampus.** (A): Schematic illustration of the untargeted metabolomic analysis. Mice were intraperitoneally injected with PBS, or 10 mg/kg/day PDA NPs (PDA) for three consecutive days (D1-D3). (B): Differential analysis graph showing significant metabolite differences between the PDA and PBS groups, measured in both positive and negative ion modes. (C): Heatmap displaying the changes in hippocampal metabolites in both the PBS and PDA groups. (D): Bar graphs (with individual data points) highlighting these metabolite changes (VIP > 1, p < 0.05). The red arrow indicates ACh levels. (E): KEGG pathway enrichment bubble diagram comparing the PBS vs. PDA groups. (F): Schematic illustration of the microdialysis procedure. Mice were intraperitoneally injected with PBS or 10 mg/kg/day PDA NPs for three consecutive days (D1-D3). (G): Bar graphs (with individual data points) showing the concentrations of DA, NE, ACh, and tyrosine in the hippocampus on D4 and D9. *P < 0.05; n = 4.

While PDA NPs increased catecholamine levels and improved cognitive function in the short term, their long-term impact was unclear. To investigate, non-targeted metabolomics was performed on hippocampal tissue from mice treated with PBS or 10 mg/kg PDA NPs 9 days post-injection (**Figure 4A**). UPLC-MS/MS identified 193 metabolites in positive mode and 176 in negative mode (**Figure S13**). In the PDA NP group, 12 metabolites increased and 10 decreased (VIP > 1, p < 0.05) (**Figure 4B**), with 9 metabolites showing consistent trends across groups. It is noteworthy that Ach levels were found to be significantly increased in the hippocampus for both PDA groups (**Figure 4C-D**). Although these metabolites were not directly linked to catecholamine neurotransmitters, they were primarily associated with pathways such as cAMP signaling and beta-alanine metabolism, suggesting that PDA NPs may enhance overall brain metabolism (**Figure 4E**).

Microdialysis in freely moving mice was used to measure hippocampal neurotransmitter levels. Mice received either PBS or 10 mg/kg/day PDA NPs for three days. On day 4, the PDA NP group showed significant increases in DA, NE, and tyrosine compared to controls, while ACh levels only trended upward. By day 9, ACh levels were significantly higher in the PDA NP group, whereas DA and tyrosine remained only slightly elevated and NE levels unexpectedly decreased compared to the PBS group (**Figure 4F-G**). These findings suggest that long-term enhancement of hippocampal ACh is not directly linked to catecholamine levels.

### Activation of MS-hippocampal projections by PDA NPs

**Figure 5:**
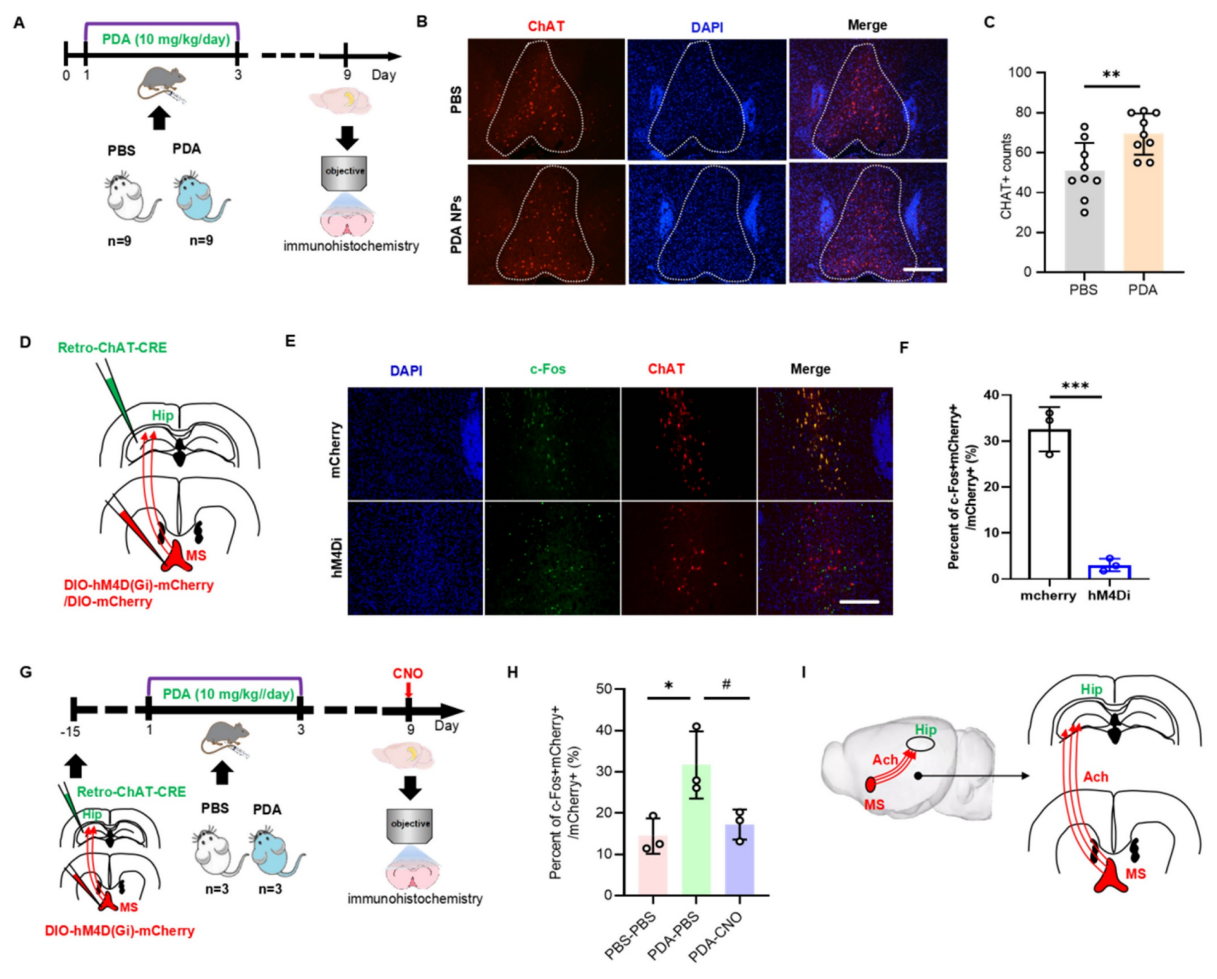
**PDA NPs activate the MS-hippocampal projections.** (A): Schematic diagram illustrating the immunofluorescence staining procedure for CHAT-positive neurons. Mice were i.p. injected with PBS (PBS group) or 10 mg/kg/day of PDA NPs (PDA group) for three consecutive days (D1-D3). (B): Representative immunofluorescence images of CHAT-positive neurons in the medial septum (MS). (C): Bar graphs (with data dots) quantifying the number of CHAT-positive cells in the MS region. **P < 0.01, n = 9. (D): Schematic diagram showing the chemogenetic inhibition of MS-projecting hippocampal neurons. In this experiment, rAAV2-retro-CHAT-Cre was injected into the hippocampus and hM4Di-DIO-mCherry (or DIO-mCherry) into the MS. (E and F): Representative images (E) and quantification (F) of c-Fos expression in the MS 90 min after CNO administration in mice transduced with mCherry or hM4Di-mCherry in the MS (scale bars, 500 μm). ***P < 0.001, n = 3. (G): Schematic diagram of chemogenetic inhibition of MS-projecting hippocampal neurons using the same viral strategy as in (D). (H): Quantification of c-Fos expression in the MS 90 min after CNO administration on D9 in mice transduced with hM4Di-mCherry in the MS (scale bars, 500 μm). *P < 0.05 (PDA-PBS vs PBS-PBS), #P < 0.05 (PDA-PBS vs PDA-CNO), n = 3. (I): Schematic illustration summarizing the mechanism, suggesting that PDA NPs selectively activate cholinergic neurotransmission from the MS to hippocampal neurons.

On day 9, hippocampal ACh levels were significantly increased in PDA NP-treated mice (**Figure 4D-G**). Since the hippocampus lacks cholinergic neurons and receives ACh from cholinergic projections originating in the medial septal nucleus (MS) 30, we investigated whether PDA NPs activate MS cholinergic neurons. Fluorescent staining for choline acetyltransferase (ChAT)—the key enzyme in ACh synthesis—in the MS region revealed enhanced red fluorescence after PDA NP treatment (**Figure 5A**), with quantification confirming increased ChAT-positive cells (**Figure 5B-C**).

To test if MS-hippocampal projections are essential for the elevated hippocampal ACh, we employed chemogenetics to selectively inhibit MS-projecting hippocampal neurons. Mice received hM4Di-DIO-mCherry or DIO-mCherry in the MS and retro-ChAT-CRE in the hippocampus, followed by i.p. of CNO before biochemical analysis (**Figure 5D**). This inhibition was validated by a marked decrease in mCherry^+^c-Fos^+^ neurons in the MS on day 9 (**Figure 5E-F**). Moreover, after PDA NP administration, inhibiting MS-projecting hippocampal neurons significantly reduced mCherry^+^c-Fos^+^ cell counts in the MS (**Figure 5G-H**). These results strongly suggest that the increased hippocampal ACh after PDA NP treatment is primarily due to activation of MS-hippocampal projections (**Figure 5I**).

### Activation of MS-hippocampal projections to release ACh by PDA NPs

**Figure 6:**
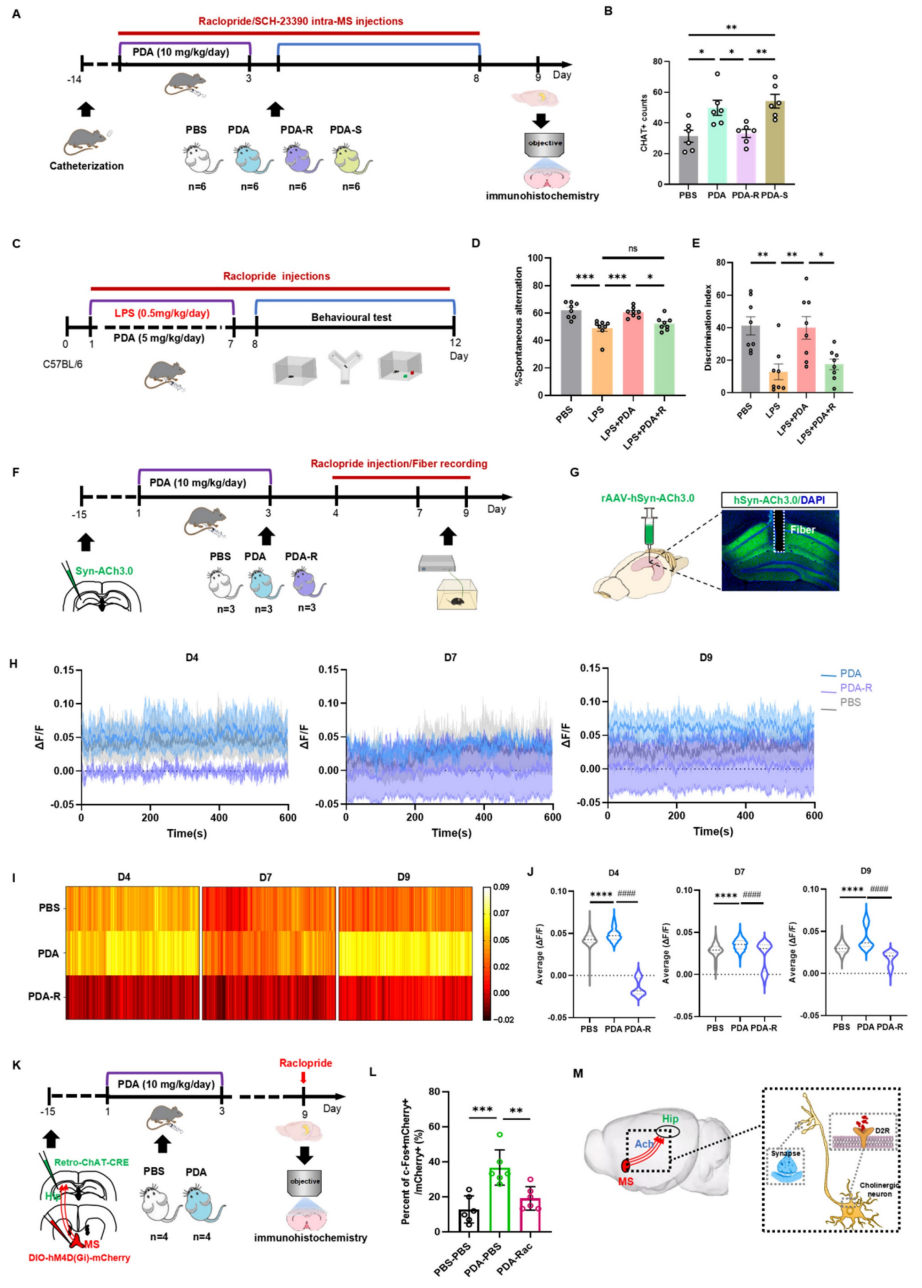
** PDA NPs activate MS-hippocampal projections via the DA D2 receptor.** (A): Schematic illustration of the experimental procedure. Mice underwent surgery and then received i.p. injections of PDA NPs (10 mg/kg/day) for three consecutive days (D1-D3). At the same time, intra-MS microinjections of raclopride or SCH23390 (0.2 μL/mouse/day) were administered on four occasions (D2, D4, D6, D8). (B): Bar graphs (with individual data points) showing the number of ChAT-positive cells in the medial septum (MS) (*P < 0.05, **P < 0.01; n = 6). (C): Schematic illustration of the experimental design. Mice were divided into four groups: one group received i.p. injections of PBS (control); the other three groups were treated with LPS followed by i.p. injections of either PBS (D1-D7; LPS group), PDA NPs (5 mg/kg/day, D1-D7; LPS+PDA group), or PDA NPs (5 mg/kg/day, D1-D7) plus raclopride (10 mg/kg/day, D1-D12; LPS+PDA-R group). Behavioral tests—including the open field test (OFT), Y-maze, and novel object recognition (NOR) test—were subsequently performed. (D): Bar graphs (with individual data points) depicting spontaneous alternation performance in the Y-maze. (E): Bar graphs (with individual data points) showing the NOR ratio in the NOR test (*P < 0.05, **P < 0.01, ***P < 0.001; n = 8). (F): Schematic diagram outlining the fiber photometry procedure for recording hippocampal ACh levels. Mice were i.p. injected with either PBS (control) or PDA NPs (10 mg/kg/day) for three consecutive days (D1-D3). An additional group received PDA NPs (10 mg/kg/day, D1-D3) followed by i.p. raclopride (10 mg/kg/day) on three occasions (D4, D7, D9) prior to recording. (G): Schematic depiction of the viral injection strategy along with representative images showing rAAV-hSyn-ACh3.0 expression in the hippocampus (scale bar: 500 μm). (H): Plots of fluorescent ACh signals recorded from the hippocampus of freely moving mice on D4, D7, and D9. (I): Heatmaps displaying the average fluorescent ACh signals in the hippocampus recorded from freely moving mice on D4, D7, and D9. (J): Bar graphs showing the average fluorescent ACh signal values in the hippocampus on D4, D7, and D9. ****P < 0.0001 (PDA vs. PBS) and ^####^P < 0.0001 (PDA vs. PDA-R), n = 5000. (K): Schematic diagram of the chemogenetic inhibition strategy for MS-projecting hippocampal neurons. rAAV2-retro-CHAT-Cre was injected into the hippocampus, and hM4Di-DIO-mCherry was injected into the MS. (L): Quantification of c-Fos expression in the MS, measured 90 min after raclopride administration on D9 in mice transduced with hM4Di-mCherry in the MS. **P < 0.01, ***P < 0.01; n = 6. (M): Schematic illustration summarizing the findings, indicating that PDA NPs selectively activate cholinergic neurotransmission from the MS to hippocampal neurons via DA D2 receptor pathways.

Given the specific expression of DA D2 receptor mRNA in the MS region 31, we next examined whether the elevated hippocampal ACh levels were mediated by the DA D2 receptor. Mice were implanted with a hippocampal cannula and divided into four groups with intra-MS injections: PBS (PBS group), PDA NPs followed by PBS (PDA group), PDA NPs followed by raclopride (a DA D2 receptor antagonist; PDA-R group), and PDA NPs followed by SCH23390 (a DA D1 receptor antagonist; PDA-S group) (**Figure 6A**). Fluorescent staining for ChAT in the MS showed that the PDA-R group had significantly fewer ChAT-positive cells compared to the PDA group, indicating that the DA D2 receptor antagonist reduced PDA NP-enhanced ACh synthesis (**Figure 6B** and **Figure S14**). Interestingly, the PDA-S group exhibited a significant increase in ChAT-positive cells compared to both the PBS and PDA-R groups, with no difference between the PDA and PDA-S groups (**Figure 6B**).

To determine whether the behavioral improvements by PDA NPs are mediated by the DA D2 receptor, LPS-treated mice were divided into four groups: one received i.p. PBS; the others received LPS followed by i.p. injections of PBS (LPS group), PDA NPs (5 mg/kg/day for days 1-7; LPS+PDA group), or PDA NPs plus raclopride (5 mg/kg/day for days 1-7 with raclopride from days 1-12; LPS+PDA-R group) (**Figure 6C**). The OFT confirmed no differences in locomotor activity (**Figure S15A**). In the Y-maze, PDA NP treatment reversed the poor spontaneous alternation seen in LPS-treated mice, but raclopride counteracted this improvement, returning performance to levels comparable to the LPS group (**Figure 6D** and **Figure S15B**). Similarly, in the NOR test, raclopride offset the elevated recognition ratios restored by PDA NPs (**Figure 6E** and **Figure S15C**), indicating that the DA D2 receptor plays a key role in the cognitive improvements observed.

Further, to assess hippocampal ACh levels and the involvement of the DA D2 receptor in PDA NP-induced ACh release, we used fiber photometry. Mice injected with rAAV-hSyn-ACh3.0 in the hippocampus were divided into three groups: one received i.p. PBS (days 1-3; PBS group), one received PDA NPs (10 mg/kg; PDA group), and one received PDA NPs plus raclopride (10 mg/kg; PDA-R group) with recordings on days 4, 7, and 9 (**Figure 6F**). During a 10-min free-moving session, the PDA group exhibited significantly higher hippocampal ACh levels on days 4, 7, and 9 compared to the PBS group (**Figure 6G-J**), consistent with previous observations (**Figure 4G**). Notably, the PDA-R group maintained significantly lower ACh levels than both the PBS and PDA groups on all days, highlighting the involvement of the DA D2 receptor. Moreover, ACh levels continued to rise over time (**Figure 6H**).

Finally, to further examine DA D2 receptor-mediated MS-hippocampal projections in regulating hippocampal ACh release, we inhibited MS-projecting hippocampal neurons using raclopride. By expressing hM4Di-DIO-mCherry (or DIO-mCherry) in the MS and retro-ChAT-CRE in the hippocampus, followed by i.p. raclopride prior to biochemical assessment, we confirmed via c-Fos labeling on day 9 that raclopride markedly reduced the number of mCherry^+^c-Fos^+^ neurons in the MS (**Figure 6K-L**).

Collectively, these findings suggest that PDA NPs selectively activate cholinergic neurotransmission from the MS to the hippocampus via DA D2 receptor pathways, thereby enhancing long-term cognitive function in mice (**Figure 6M**).

## Discussion

The major findings of this study are as follows: First, PDA NPs were fabricated via the oxidative self-polymerization reaction from DA hydrochloride, resulting in uniformly spherical particles (~250 nm diameter) with a highly negative surface charge (-43.7 mV) and excellent biocompatibility. Second, using surrogate fluorescently labeled NPs, the study showed that PDA NPs cross the BBB efficiently via endocytosis, with *in vivo* imaging confirming their presence in the brain. Third, in an acidic environment (pH 4), PDA NPs degrade, releasing DA and forming smaller granules. This controlled release was confirmed by UV-Vis absorption, HPLC, and TEM imaging. Fourth, PDA NPs are internalized by endothelial and neuronal cells. Once inside acidic vesicles, they degrade to release DA, which can then be converted into other catecholamines like NE. Fifth, in a mouse model of LPS-induced cognitive deficits, treatment with PDA NPs improved performance in behavioral tests (Y-maze and NOR), indicating alleviation of cognitive impairments. Sixth, short-term treatment increased levels of DA, NE, and tyrosine, while long-term effects were characterized by a significant elevation in hippocampal ACh. This enhancement of ACh was linked to the activation of cholinergic projections from the MS. Finally, pharmacological and chemogenetic experiments revealed that the PDA NP-induced increase in ACh—and the associated cognitive benefits—is mediated by DA D2 receptor pathways in the MS, highlighting a critical mechanism for modulating hippocampal cholinergic activity. In summary, the study provides compelling evidence that PDA NPs serve as a promising, carrier-free platform for exogenous catecholamine delivery, improving cognitive deficits by modulating both dopaminergic and cholinergic pathways.

Although PDA NPs have been widely applied in medicine, detailed investigations into the structure of PDA were limited until 2012. Two studies that year proposed that PDA is a supramolecular aggregate of monomers (such as DA chrome), assembled via noncovalent interactions, including physical trimers of (DA)₂/DHI 32,21. This model suggests that PDA may exert neuroprotective effects through the release of DA. However, the roles of DHI and other DA derivatives in cognitive enhancement remain unclear. Some researchers have noted similarities between PDA and neuromelanin in terms of synthesis and molecular structure 33,34, yet the *in vivo* mechanisms and metabolites of neuromelanin are not fully understood 35,36. Addressing these gaps, our research focused on the mechanisms by which PDA NPs enhance cognitive learning.

Our previous work showed that intraperitoneally administered PDA NPs distribute to the heart, liver, spleen, lungs, kidneys, and brain in mice 18. In the present study, we sought to determine whether PDA improves cognitive performance by releasing DA within the brain. To verify brain entry, we (i) directly detected PDA NPs in brain sections and (ii) tracked fluorescence from PDA-simulated fluorescent particles after systemic administration; both approaches demonstrated robust brain signals, confirming NP presence. Because DA and ACh do not cross the BBB in physiologically meaningful amounts, peripheral pools are unlikely to account for the observed elevations in brain DA and ACh. Thus, the increased central neurotransmitter levels most likely reflect the action of brain-penetrant PDA NPs. These findings provide a mechanistic basis for the subsequent behavioral effects and lay the groundwork for designing region-targeted PDA NP delivery systems, particularly for septo-hippocampal circuits.

In co-culture experiments with PDA NPs and bend.3 endothelial cells, PDA NPs localized in vesicles at a pH of around 5.6—typical of endosomes and lysosomes—indicating that PDA NPs degrade in this acidic environment 37. Moreover, NPs are frequently reported to be preferentially internalized by immune cells *in vivo*. To examine cell-type specificity under controlled conditions, we co-cultured PDA NPs with BRL-3A hepatocytes, PC12 neuronal-like cells, and RAW 264.7 macrophages. As expected, RAW 264.7 cells exhibited markedly greater NP uptake than the other two cell types (**Figure S16**). Moreover, we observed stable levels of DA and tyrosine in the culture supernatant, while NE levels increased significantly. Endothelial cells are known to express key enzymes—tyrosine hydroxylase, dopa decarboxylase, DA β-hydroxylase (DβH), and phenylethanolamine- N-methyltransferase—essential for catecholamine synthesis and release 38,39. This suggests two possible mechanisms: either PDA NPs enter intracellular vesicles and release DA that is then converted to NE via DβH, or PDA NPs stimulate the overall catecholamine system. Given that DA and tyrosine levels remained unchanged, our data support the hypothesis that the increase in NE is mainly due to DA conversion by DβH. Further research is needed to confirm these mechanisms.

Abnormal catecholaminergic neurotransmission is a key contributor to neurodegenerative diseases characterized by cognitive impairment, such as AD and PD 1,2. Inflammation has been shown to disrupt catecholamine signaling 40,41. Accordingly, we employed an LPS-induced systemic inflammation model to elicit cognitive impairment in mice. Consistent with this rationale, hippocampal catecholamine metabolites were significantly reduced in LPS-treated mice (**Figure 3H**). Although PDA NPs are polymerized from DA and are widely used for their antioxidant and free-radical-scavenging properties, our study focused on their effects on neurocognitive function via catecholaminergic signaling. In LPS-treated mice, PDA NP administration improved cognitive performance, coinciding with the release of dopamine-like catecholamines and increased hippocampal ACh. At the same time, the abundant phenolic hydroxyl groups in PDA confer strong oxygen radical-scavenging activity (**Figure S17**). Therefore, we cannot exclude contributions from anti-inflammatory and ROS-scavenging mechanisms, which merit further investigation.

A major novel finding of our study is that PDA NPs increase ACh levels in the hippocampus—a neurotransmitter critical for memory and learning 42,43. Although the precise mechanism by which PDA NPs activate cholinergic neurons in the MS was not fully delineated, previous studies indicate that these neurons express the DA D5 receptor, a subtype of the D1 receptor family 44. Activation of DA D1 receptors is known to elevate cyclic adenosine monophosphate (cAMP) levels, which in turn promotes neurotransmitter release 45. Based on our results and related studies 46, we deduce that DA released from PDA NPs may stimulate DA D2 receptors in the MS, leading to enhanced ACh release via cholinergic projections to the hippocampus.

DA is essential for regulating mood, motivation, attention, and learning, and deficits in DA signaling are linked to various cognitive impairments 47,48. Although levodopa (L-DOPA) is commonly used to treat Parkinson's disease, its clinical use is hampered by issues related to BBB permeability and side effects. In this context, PDA NPs may offer a promising alternative. Our data indicate that while hippocampal DA levels decline over time following PDA NP administration, hippocampal ACh levels increase, leading to improved cognitive behavior in both the short and long term. Moreover, the administration of the DA D2 antagonist raclopride reduced hippocampal ACh levels in a DA-dependent manner, suggesting that short-term cognitive improvements are primarily due to increased DA, whereas long-term benefits arise from enhanced cholinergic signaling via MS-hippocampal projections mediated by DA D2 receptors.

Although best known for their roles in the nervous system, peripheral levels of NE, ACh, and tyrosine have broad physiological effects. As an α1-adrenergic receptor agonist, NE elevates systolic, diastolic, and pulse pressures and produces positive inotropy; when excessive, peripheral NE can contribute to local tissue injury (e.g., via intense vasoconstriction) 49. NE also modulates glucose metabolism and immune responses, with implications for obesity and autoimmunity 50,51. ACh participates widely in peripheral physiology—including regulation of cardiac function and blood pressure, intestinal peristalsis, and glandular secretion—and engages cholinergic pathways that influence immunity and inflammation 52,53. Tyrosine, the biosynthetic precursor of DA and NE, affects catecholamine synthesis in peripheral tissues and thereby influences stress responses, metabolic processes (fat and carbohydrate metabolism), and immune functions 54,55. Collectively, fluctuations in peripheral NE, ACh, and tyrosine can impact cardiovascular, metabolic, and immune homeostasis. Accordingly, dosing and targeting strategies for drug administration are critical to harness central benefits while limiting peripheral effects.

We observed minimal tissue toxicity of PDA NPs in major organs—including brain, heart, liver, spleen, lung, and kidney—both at 9 days and 60 days after i.p. administration (**Figure S18 and Figure S19**). Prior studies report that PDA NPs undergo complete degradation by ~60 days 56 and are rapidly cleared via the gastrointestinal tract within 24 h 57. In our metabolomics analysis, PDA degradation products were detectable at day 3 but not at day 9. Notably, high concentrations (100 µg/mL) of PDA NPs reduced viability in several cell lines (**Figure S20**). Together, these findings indicate that therapeutic efficacy and safety are dose dependent, underscoring the need to optimize dosing regimens in future studies.

This study has several limitations. First, our focus was on short- to medium-term effects (up to nine days), leaving the long-term efficacy and safety of PDA NPs unaddressed. Second, although our data suggest that PDA NPs improve cognitive function by promoting DA release and activating cholinergic neurons, the detailed molecular mechanisms—particularly the roles of different DA receptor subtypes—remain unclear. Third, the potential contributions of the antioxidant and anti-inflammatory properties of PDA NPs were not fully separated from their neurotransmitter-related effects. Future studies should address these limitations and further evaluate the safety profiles.

**Figure 7:**
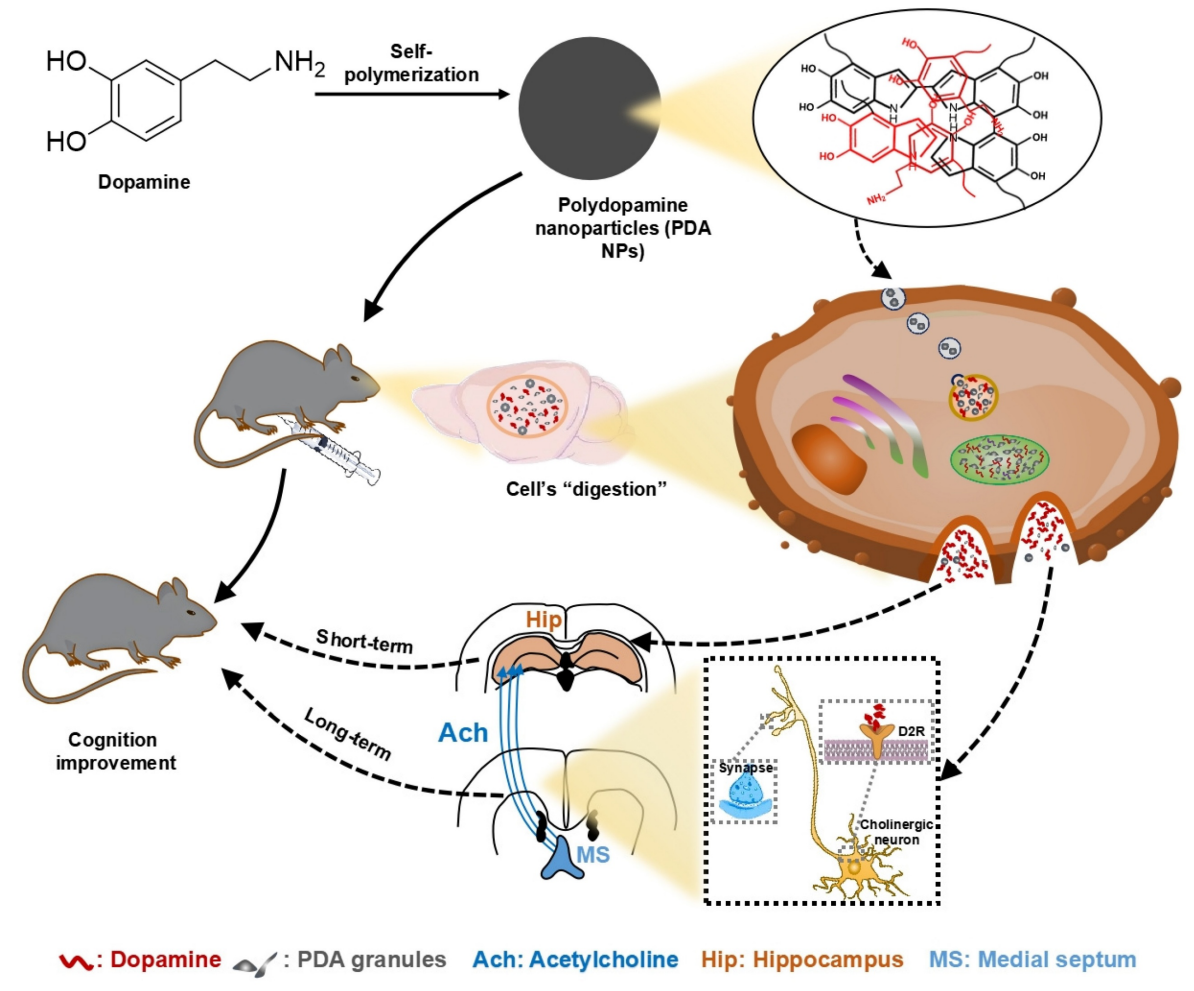
** Proposed mechanisms for the beneficial impact of PDA NPs on cognitive function.** After systemic administration, PDA NPs cross the BBB and are subsequently degraded by cells, influencing brain function. In the short term, PDA NPs elevate brain dopamine (DA) levels, which improves cognitive performance. In the long term, PDA NPs enhance hippocampal acetylcholine (ACh) levels by activating cholinergic projections from the medial septal nucleus (MS) to the hippocampus, partly through DA D2 receptor-mediated pathways.

In conclusion, this study demonstrates that PDA NPs effectively cross the BBB and release DA, thereby improving cognitive deficits in LPS-treated mice. In the short term, PDA NP administration boosts hippocampal DA levels, while long-term treatment enhances hippocampal ACh via activation of medial septal-hippocampal cholinergic projections mediated by DA D2 receptors (**Figure 7**). These findings position PDA NPs as a promising nanotherapeutic strategy for treating cognitive impairments associated with psychiatric and neurological disorders.

## Supplementary Material

Supplementary figures.

## Figures and Tables

**Table 1:** Detection parameters for each molecular

Compound	Parention	Quantitative ion	Detection mode	Cone-hole voltage V	Collision energy V
DA	154.2	91.3	[M+H]^+^	20	31
ACh	146.3	87.1	[M+H]^+^	26	21
NE	152.0	135.0	[M+H]^+^	25	12
Try	180.07	163.04	[M-H]^-^	30	27

## References

[1] Youdim MB, Fridkin M, Zheng H (2005). Bifunctional drug derivatives of MAO-B inhibitor rasagiline and iron chelator VK-28 as a more effective approach to treatment of brain ageing and ageing neurodegenerative diseases. Mech Ageing Dev.

[2] Caligiore D, Silvetti M, D'Amelio M, Puglisi-Allegra S, Baldassarre G (2020). Computational Modeling of Catecholamines Dysfunction in Alzheimer's Disease at Pre-Plaque Stage. J Alzheimers Dis.

[3] Delgado PL (2000). Depression: the case for a monoamine deficiency. J Clin Psychiatry.

[4] Abbott NJ, Patabendige AA, Dolman DE, Yusof SR, Begley DJ (2010). Structure and function of the blood-brain barrier. Neurobiol Dis.

[5] Pardridge WM (2005). The blood-brain barrier: bottleneck in brain drug development. NeuroRx.

[6] Pardridge WM (2007). Blood-brain barrier delivery. Drug Discov Today.

[7] Tsuji A (2005). Small molecular drug transfer across the blood-brain barrier via carrier-mediated transport systems. NeuroRx.

[8] Wang Z, Duan Y, Duan Y (2018). Application of polydopamine in tumor targeted drug delivery system and its drug release behavior. J Control Release.

[9] Xu X, Wang L, Luo Z, Ni Y, Sun H, Gao X (2017). Facile and Versatile Strategy for Construction of Anti-Inflammatory and Antibacterial Surfaces with Polydopamine-Mediated Liposomes Releasing Dexamethasone and Minocycline for Potential Implant Applications. ACS Appl Mater Interfaces.

[10] Yang Z, Huang R, Zheng B, Guo W, Li C, He W (2021). Highly Stretchable, Adhesive, Biocompatible, and Antibacterial Hydrogel Dressings for Wound Healing. Adv Sci (Weinh).

[11] Choi CKK, Choi CHJ, Bian L (2015). A gold@polydopamine core-shell nanoprobe for long-term intracellular detection of microRNAs in differentiating stem cells. J Am Chem Soc.

[12] Lin LS, Cong ZX, Cao JB, Ke KM, Peng QL, Gao J (2014). Multifunctional Fe₃O₄@polydopamine core-shell nanocomposites for intracellular mRNA detection and imaging-guided photothermal therapy. ACS Nano.

[13] Lee H, Dellatore SM, Miller WM, Messersmith PB (2007). Mussel-inspired surface chemistry for multifunctional coatings. Science.

[14] Liu X, Cao J, Li H, Li J, Jin Q, Ren K (2013). Mussel-inspired polydopamine: a biocompatible and ultrastable coating for nanoparticles in vivo. ACS Nano.

[15] Ju KY, Lee Y, Lee S, Park SB, Lee JK (2011). Bioinspired polymerization of dopamine to generate melanin-like nanoparticles having an excellent free-radical-scavenging property. Biomacromolecules.

[16] Bao X, Zhao J, Sun J, Hu M, Yang X (2018). Polydopamine Nanoparticles as Efficient Scavengers for Reactive Oxygen Species in Periodontal Disease. ACS Nano.

[17] Li J, Hou W, Lin S, Wang L, Pan C, Wu F (2022). Polydopamine Nanoparticle-Mediated Dopaminergic Immunoregulation in Colitis. Adv Sci (Weinh).

[18] Zhu TT, Wang H, Gu HW, Ju LS, Wu XM, Pan WT, Zhao MM (2023). Melanin-like polydopamine nanoparticles mediating anti-inflammatory and rescuing synaptic loss for inflammatory depression therapy. J Nanobiotechnology.

[19] Liu Y, Ai K, Ji X, Askhatova D, Du R, Lu L (2017). Comprehensive Insights into the Multi-Antioxidative Mechanisms of Melanin Nanoparticles and Their Application To Protect Brain from Injury in Ischemic Stroke. J Am Chem Soc.

[20] d'Ischia M, Napolitano A, Ball V, Chen CT, Buehler MJ (2014). Polydopamine and eumelanin: from structure-property relationships to a unified tailoring strategy. Acc Chem Res.

[21] Hong S, Na YS, Choi S, Song IT, Kim WY, Lee H (2012). Non-covalent self-assembly and covalent polymerization co-contribute to polydopamine formation. Adv Fun Mater.

[22] Ding L, Zhu X, Wang Y, Shi B, Ling X, Chen H (2017). Intracellular Fate of Nanoparticles with Polydopamine Surface Engineering and a Novel Strategy for Exocytosis-Inhibiting, Lysosome Impairment-Based Cancer Therapy. Nano Lett.

[23] Zhao J, Bi W, Xiao S, Lan X, Cheng X, Zhang J (2019). Neuroinflammation induced by lipopolysaccharide causes cognitive impairment in mice. Sci Rep.

[24] Xiao M, Li Y, Allen MC, Deheyn DD, Yue X, Zhao J (2015). Bio-Inspired Structural Colors Produced via Self-Assembly of Synthetic Melanin Nanoparticles. ACS Nano.

[25] Ding F, Gao X, Huang X, Ge H, Xie M, Qian J (2020). Polydopamine-coated nucleic acid nanogel for siRNA-mediated low-temperature photothermal therapy. Biomaterials.

[26] Borovanský J, Elleder M (2003). Melanosome degradation: fact or fiction. Pigment Cell Res.

[27] Furtado D, Björnmalm M, Ayton S, Bush AI, Kempe K, Caruso F (2018). Overcoming the Blood-Brain Barrier: The Role of Nanomaterials in Treating Neurological Diseases. Adv Mater.

[28] Patel S, Kim J, Herrera M, Mukherjee A, Kabanov AV, Sahay G (2019). Brief update on endocytosis of nanomedicines. Adv Drug Deliv Rev.

[29] Rush RA, Geffen LB (1980). Dopamine beta-hydroxylase in health and disease. Crit Rev Clin Lab Sci.

[30] Wang Y, Wang Y, Xu C, Wang S, Tan N, Chen C (2020). Direct Septum-Hippocampus Cholinergic Circuit Attenuates Seizure Through Driving Somatostatin Inhibition. Biol Psychiatry.

[31] Weiner DM, Levey AI, Sunahara RK, Niznik HB, O'Dowd BF, Seeman P (1991). D1 and D2 dopamine receptor mRNA in rat brain. Proc Natl Acad Sci U S A.

[32] Dreyer DR, Miller DJ, Freeman BD, Paul DR, Bielawski CW (2012). Elucidating the structure of poly(dopamine). Langmuir.

[33] d'Ischia M, Wakamatsu K, Napolitano A, Briganti S, Garcia-Borron JC, Kovacs D (2013). Melanins and melanogenesis: methods, standards, protocols. Pigment Cell Melanoma Res.

[34] Ito S (2006). Encapsulation of a reactive core in neuromelanin. Proc Natl Acad Sci U S A.

[35] Nagatsu T, Nakashima A, Watanabe H, Ito S, Wakamatsu K (2022). Neuromelanin in Parkinson's Disease: Tyrosine Hydroxylase and Tyrosinase. Int J Mol Sci.

[36] Vila M (2019). Neuromelanin, aging, and neuronal vulnerability in Parkinson's disease. Mov Disord.

[37] Gros F, Muller S (2023). The role of lysosomes in metabolic and autoimmune diseases. Nat Rev Nephrol.

[38] Pfeil U, Kuncova J, Brüggmann D, Paddenberg R, Rafiq A, Henrich M (2014). Intrinsic vascular dopamine - a key modulator of hypoxia-induced vasodilatation in splanchnic vessels. J Physiol.

[39] Sorriento D, Santulli G, Del Giudice C, Anastasio A, Trimarco B, Iaccarino G (2012). Endothelial cells are able to synthesize and release catecholamines both in vitro and in vivo. Hypertension.

[40] Morella IM, Brambilla R, Morè L (2022). Emerging roles of brain metabolism in cognitive impairment and neuropsychiatric disorders. Neurosci Biobehav Rev.

[41] Feng Y, Lu Y (2021). Immunomodulatory Effects of Dopamine in Inflammatory Diseases. Front Immunol.

[42] Hasselmo ME (2006). The role of acetylcholine in learning and memory. Curr Opin Neurobiol.

[43] Haam J, Yakel JL (2017). Cholinergic modulation of the hippocampal region and memory function. J Neurochem.

[44] Berlanga ML, Simpson TK, Alcantara AA (2005). Dopamine D5 receptor localization on cholinergic neurons of the rat forebrain and diencephalon: a potential neuroanatomical substrate involved in mediating dopaminergic influences on acetylcholine release. J Comp Neurol.

[45] Price CJ, Kim P, Raymond LA (1999). D1 dopamine receptor-induced cyclic AMP-dependent protein kinase phosphorylation and potentiation of striatal glutamate receptors. J Neurochem.

[46] Yin L, Zhang J, Ma H, Zhang X, Fan Z, Yang Y, Li M (2023). Selective activation of cholinergic neurotransmission from the medial septal nucleus to hippocampal pyramidal neurones improves sepsis-induced cognitive deficits in mice. Br J Anaesth.

[47] Millan MJ, Agid Y, Brüne M, Bullmore ET, Carter CS, Clayton NS (2012). Cognitive dysfunction in psychiatric disorders: characteristics, causes and the quest for improved therapy. Nat Rev Drug Discov.

[48] Aarsland D, Batzu L, Halliday GM, Geurtsen GJ, Ballard C, Ray Chaudhuri K (2021). Parkinson disease-associated cognitive impairment. Nat Rev Dis Primers.

[49] García-Uribe J, Lopera-Jaramillo D, Gutiérrez-Vargas J, Arteaga-Noriega A, Bedoya OA (2023). Adverse effects related with norepinephrine through short peripheral venous access: Scoping review. Enferm Intensiva (Engl Ed).

[50] Sharma D, Farrar JD (2020). Adrenergic regulation of immune cell function and inflammation. Semin Immunopathol.

[51] Nonogaki K (2000). New insights into sympathetic regulation of glucose and fat metabolism. Diabetologia.

[52] Van der Zee EA, Platt B, Riedel G (2011). Acetylcholine: future research and perspectives. Behav Brain Res.

[53] Cox MA, Bassi C, Saunders ME, Nechanitzky R, Morgado-Palacin I, Zheng C (2020). Beyond neurotransmission: acetylcholine in immunity and inflammation. J Intern Med.

[54] Tank AW, Lee Wong D (2015). Peripheral and central effects of circulating catecholamines. Compr Physiol.

[55] Ma C, Dan Y (2025). The how and why of sleep: Motor theory and catecholamine hypothesis. Neuron.

[56] Bettinger CJ, Bruggeman JP, Misra A, Borenstein JT, Langer R (2009). Biocompatibility of biodegradable semiconducting melanin films for nerve tissue engineering. Biomaterials.

[57] Li J, Wang T, Kirtane AR, Shi Y, Jones A, Moussa Z (2020). Gastrointestinal synthetic epithelial linings. Sci Transl Med.

