# Supplementary material for: Polydopamine nanoparticles restore cognition via targeted dopamine delivery and septo-hippocampal cholinergic activation
Source: Theranostics. 2026 Jan 1;16(7):3616–33. doi: 10.7150/thno.121735 (PMC12846747; doi:10.7150/thno.121735)
Supplement: Supplementary file 1 — Supplementary figures. [file thnov16p3616s1.pdf]

Supplementary Materials for

Polydopamine nanoparticles restore cognition via targeted dopamine delivery and septo-hippocampal cholinergic activation

Pan-Miao Liu *et al.*

*Corresponding authors:
Dr. Jian-Jun Yang (e-mail: yjyangjj@zzu.edu.cn)
Dr. Kenji Hashimoto (e-mail: hashimoto@faculty.chiba-u.jp).

This PDF file includes:

Supplementary Text
Fig. S1 to S20

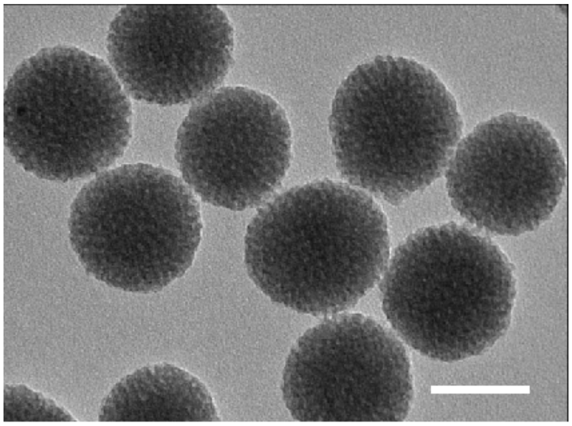

Figure. S1. TEM image of MSN. Scale bar: 200 nm

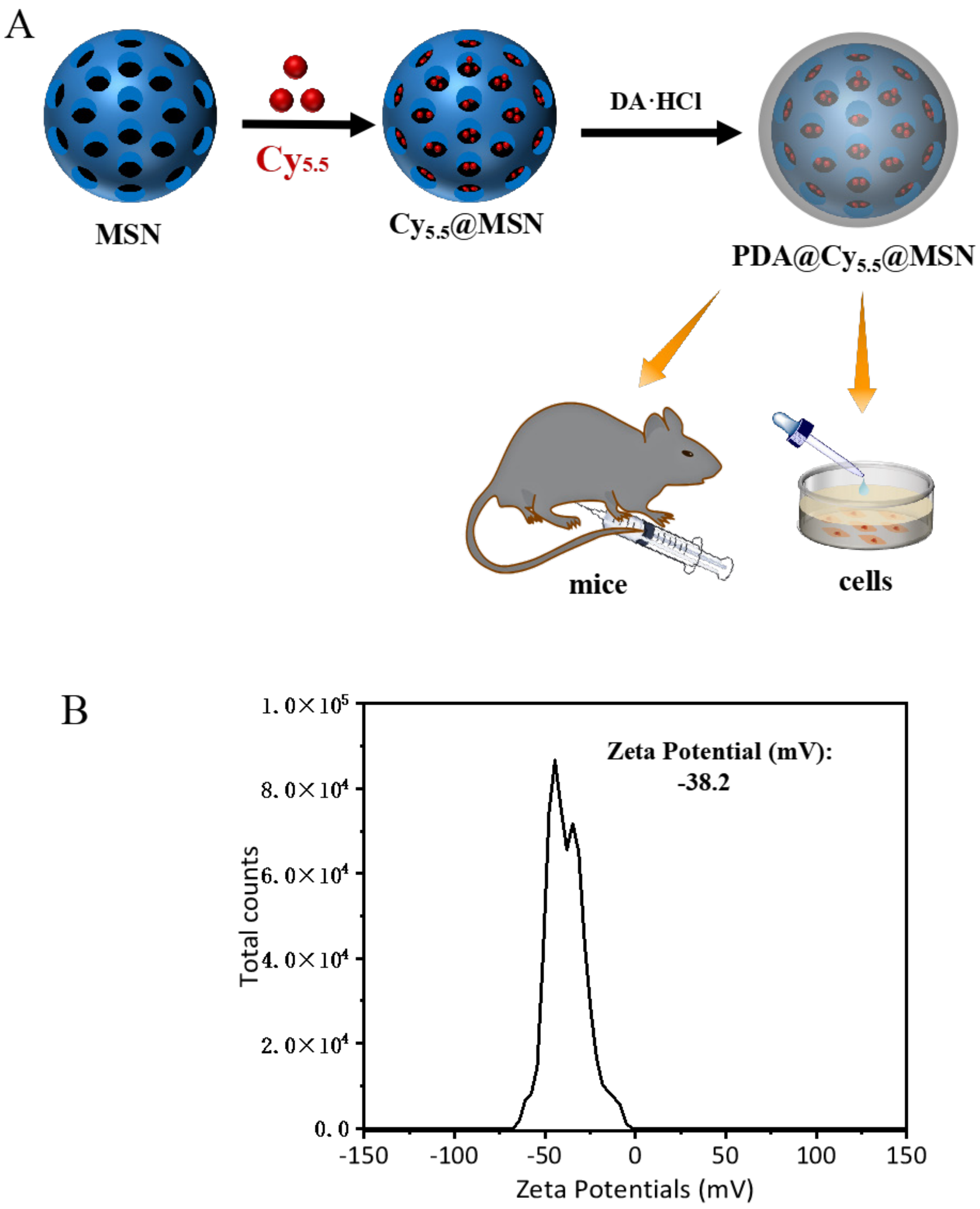

Figure. S2. (A): Schematic diagram illustrating the synthesis of PDA@$Cy_{5.5}$@MSN and subsequent processing steps. (B): Zeta potentials analysis of PDA@$Cy_{5.5}$@MSN.

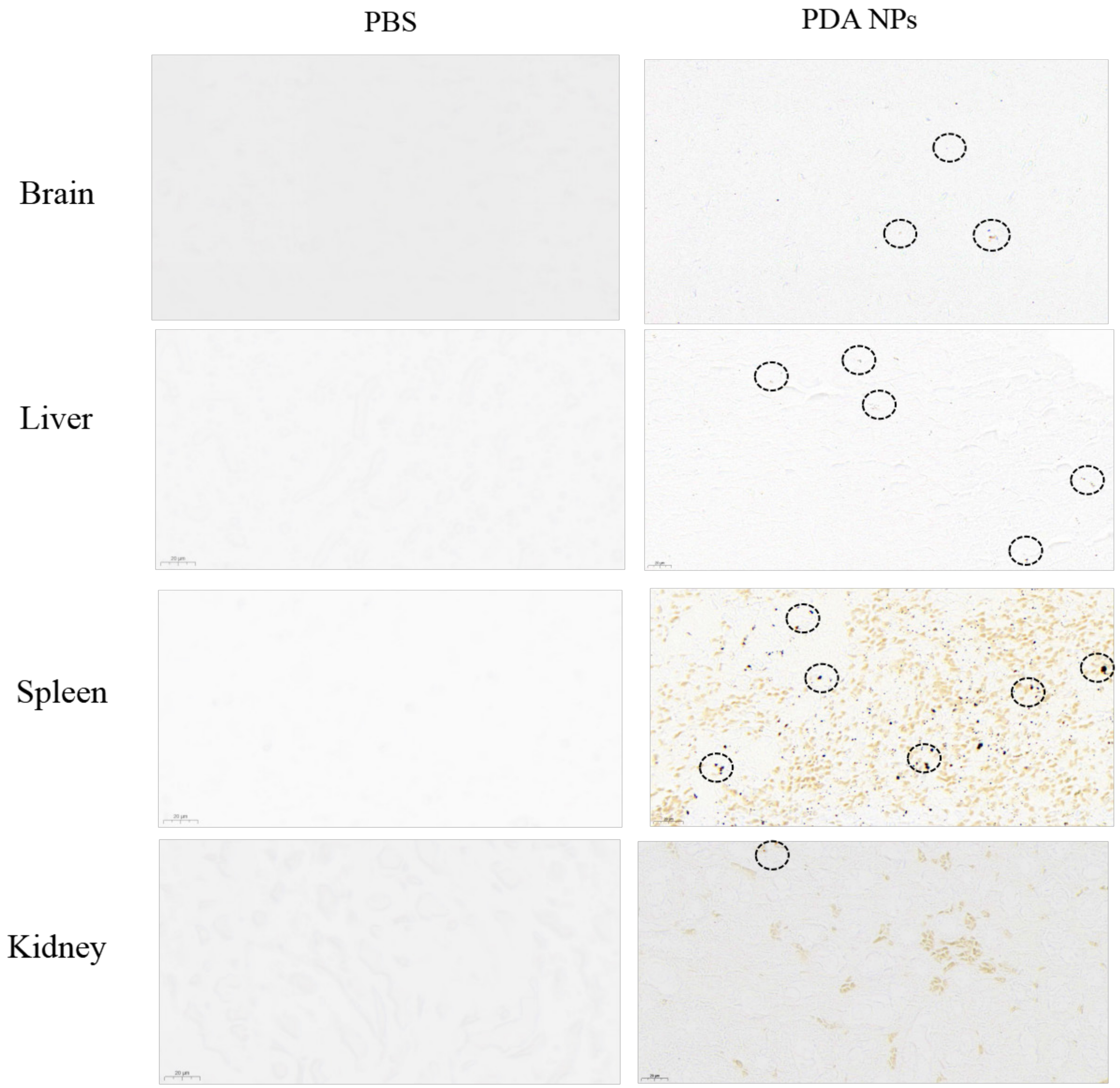

Figure. S3. Optical photograph of brain, liver, spleen and kidney in the PBS-treated and PDA NPs-treated mice. Scale bar: 20 μm.

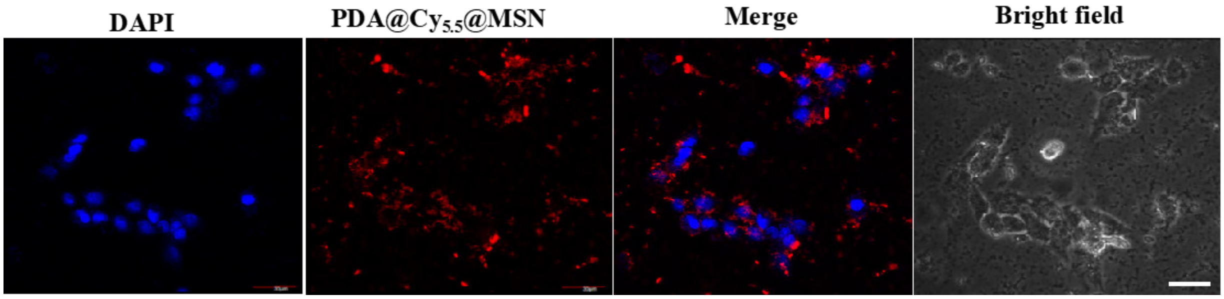

Figure. S4. Microscopy images showing PDA@Cy5.5@MSN after 1 hour of co-culture with HeLa cells. Scale bar: 30 μm.

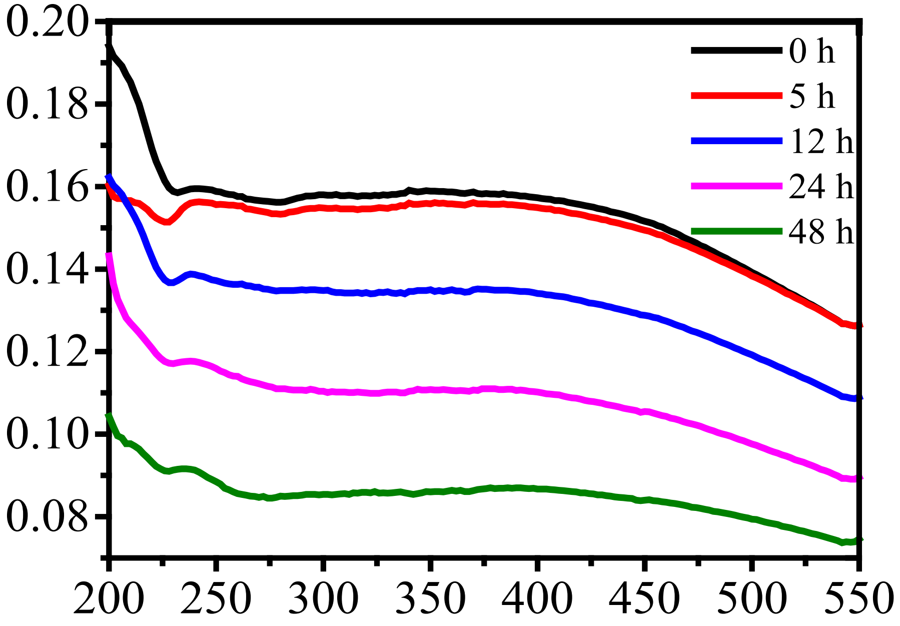

Figure. S5. Ultraviolet-visible absorption spectrum of PDA NPs, showing a decrease in absorption in the 200–550 nm range in acidic conditions.

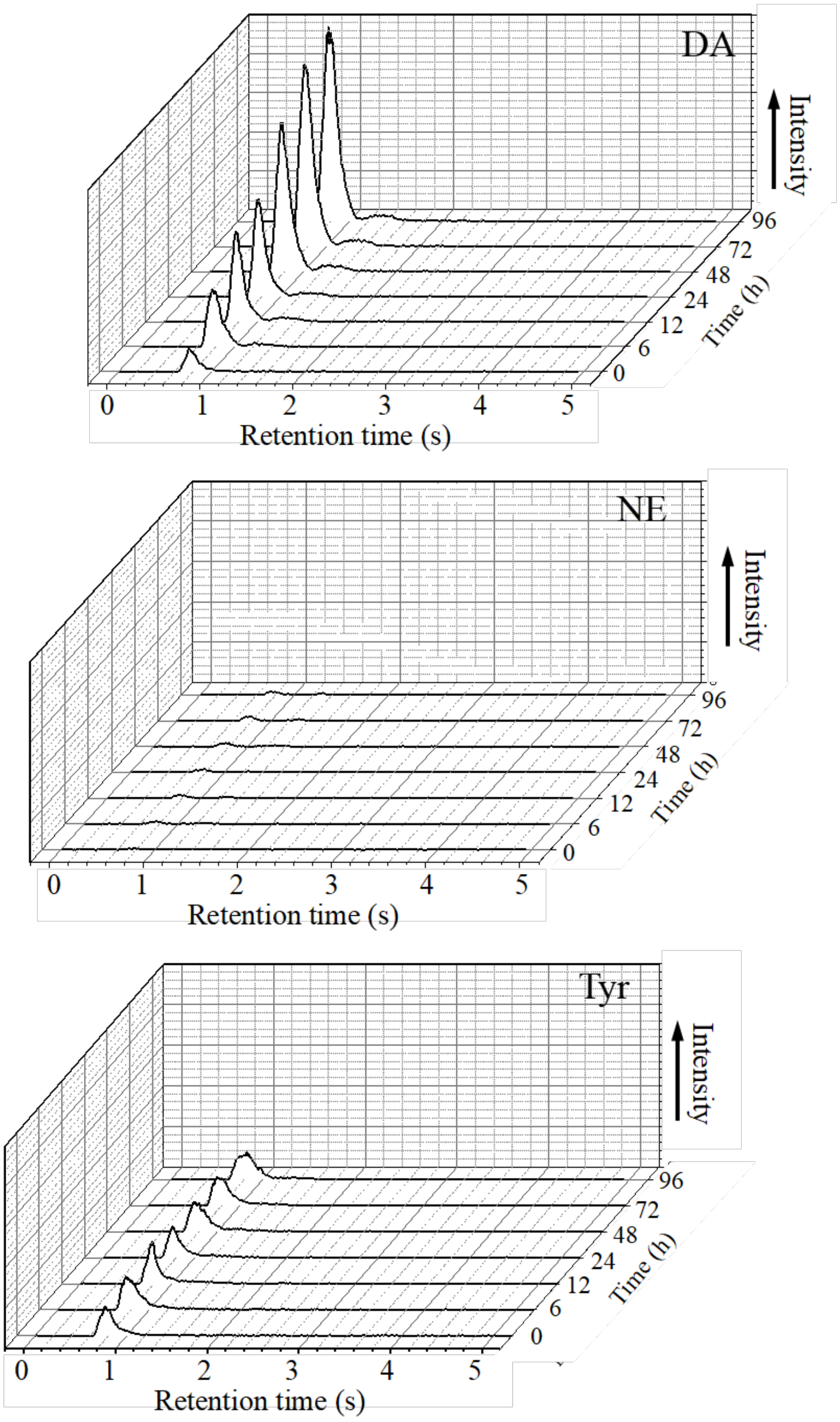

Figure. S6. HPLC chromatograms displaying the concentrations of DA, NE, and tyrosine during the co-culture of PDA NPs with bend.3 cells at various time points (0, 6, 12, 24, 48, 72, and 96 hours).

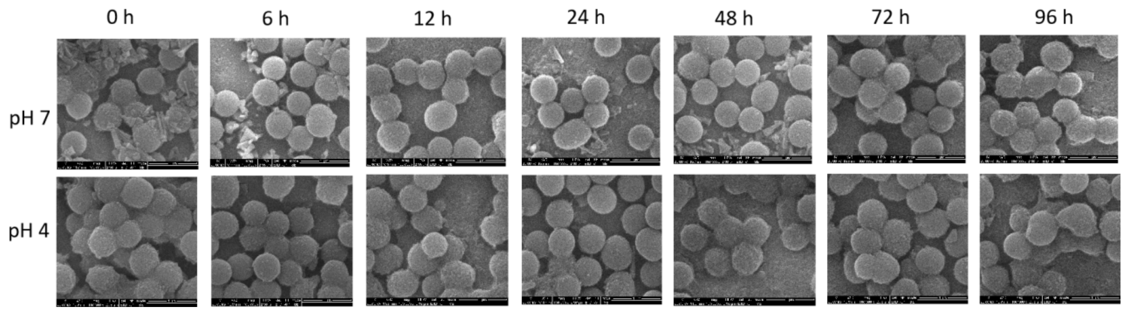

Figure. S7. SEM images of PDA NPs at various time points (0, 6, 12, 24, 48, 72, and 96 hours) after being placed in solutions with pH 4 and pH 7. Scale bar: 50 nm.

A

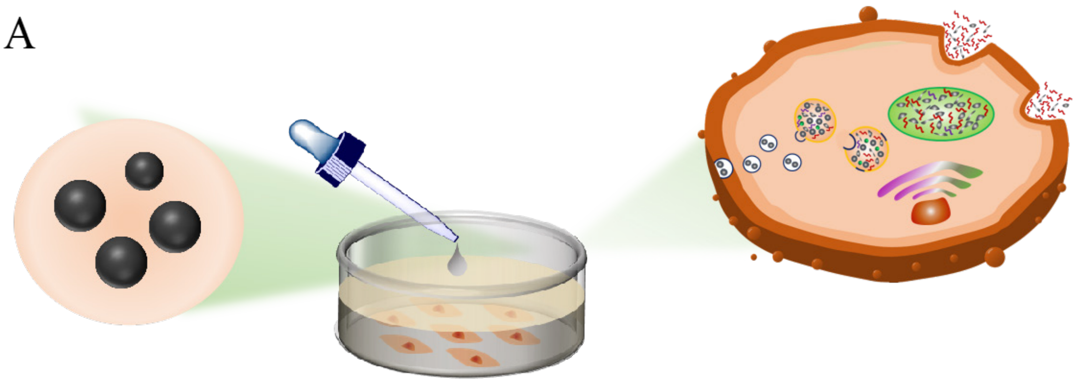

B

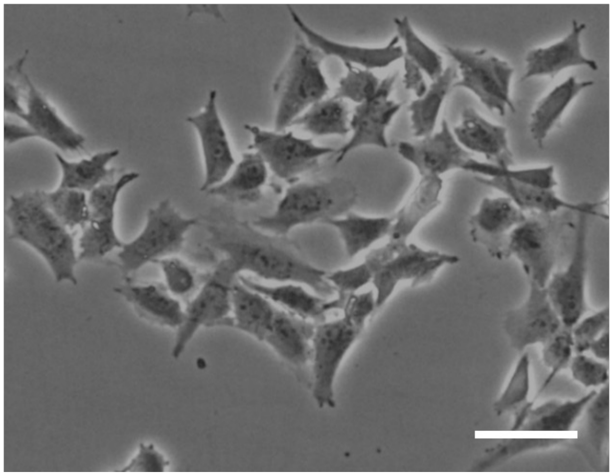

Figure. S8. (A): Schematic illustrating the co-culture of PDA NPs with cells. (B): Optical photograph of bend.3 cells after 12 hours of co-culture with PDA NPs (scale bar: 20 μm).

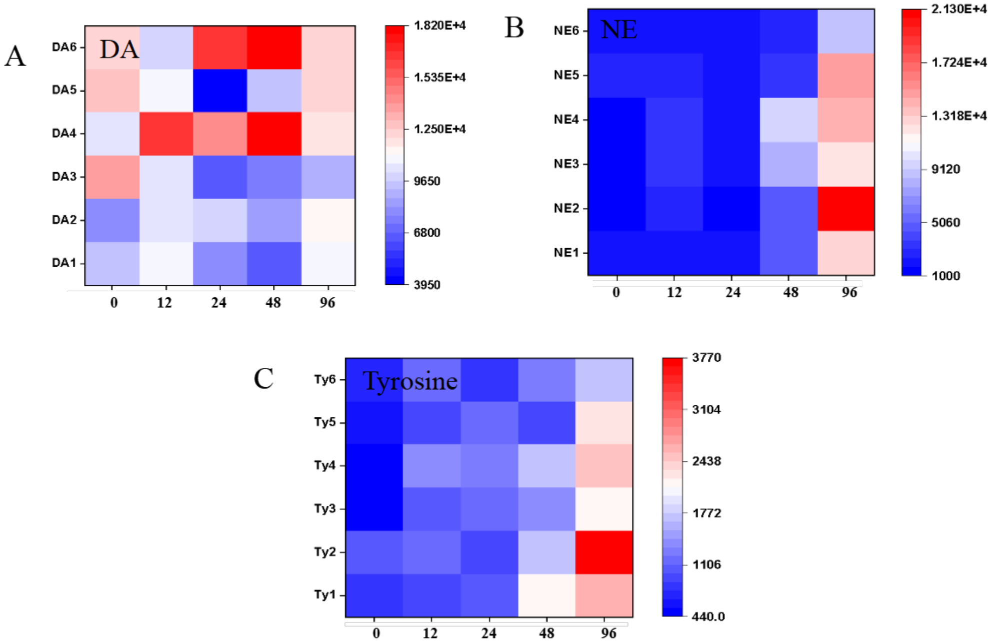

Figure. S9. Heatmap depicting the relative levels of dopamine (DA), norepinephrine (NE), and tyrosine over time (0, 12, 24, 48, and 96 hours) during co-culture of PDA NPs with Bend.3 cells.

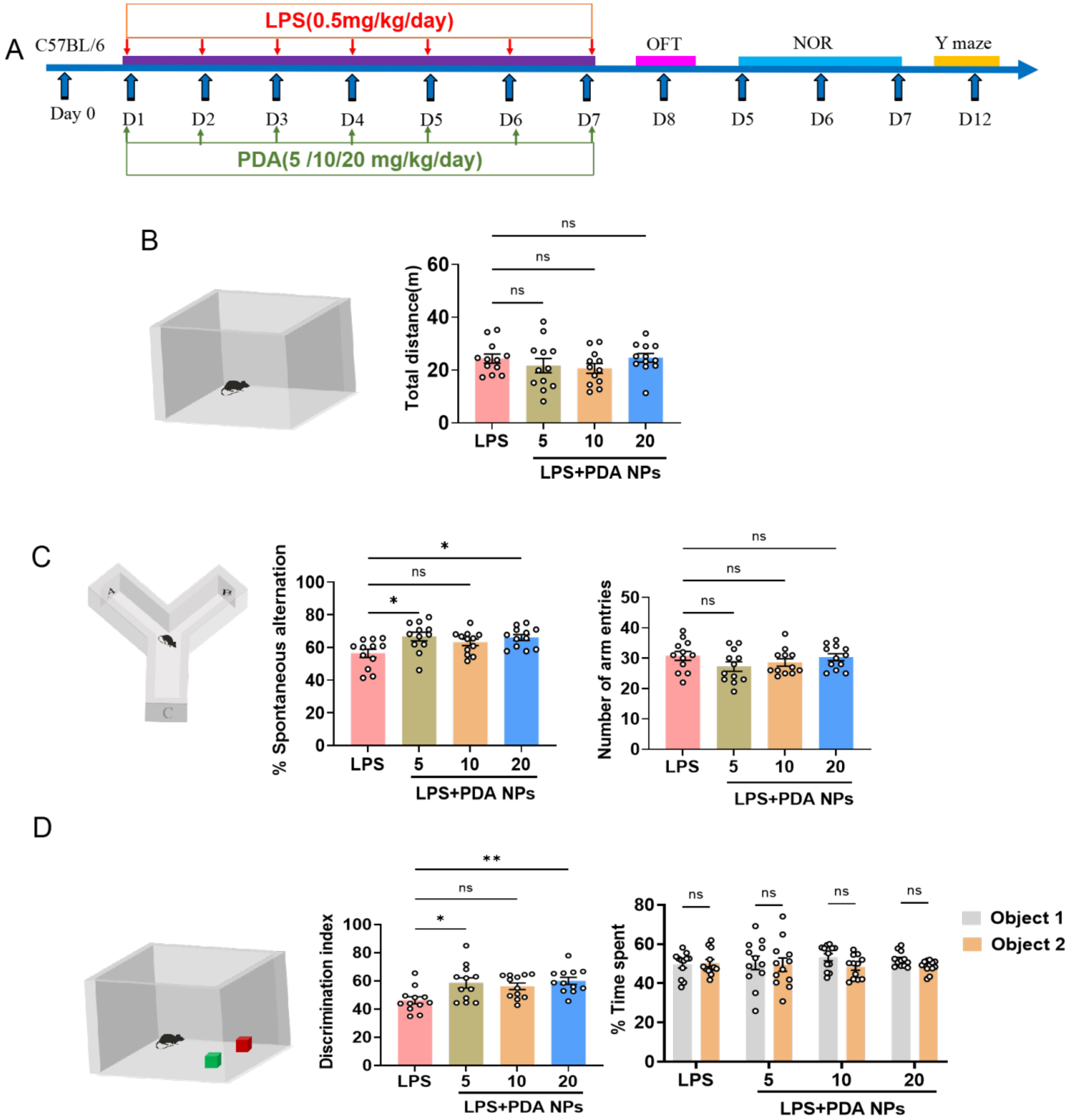

Figure. S10. **Effects of PDA NPs on cognitive deficits in LPS-treated mice.** (A): Schematic illustration of the experimental procedure. Mice were intraperitoneally (i.p.) injected with LPS (0.5 mg/kg/day) for seven consecutive days (D1–D7) to induce cognitive deficits. Concurrently, PDA NPs (5, 10, or 20 mg/kg/day) were administered i.p. for seven consecutive days (D1–D7). Behaviors tests including the open field test (OFT), Y-maze, and novel object recognition (NOR) test were conducted. (B): Bar graphs with dots depict the total distances traveled during the 5-minute duration of the OFT. (C): Bar graphs with dots illustrate the spontaneous alternation behavior and the number of arm entries in the Y-maze. (D): Bar graphs with dots display the novel object recognition ratio performance, along with the time spent interacting with object 1 and object 2 during the training section in the NOR test. *$P < 0.05$, **$P < 0.01$, n=12.

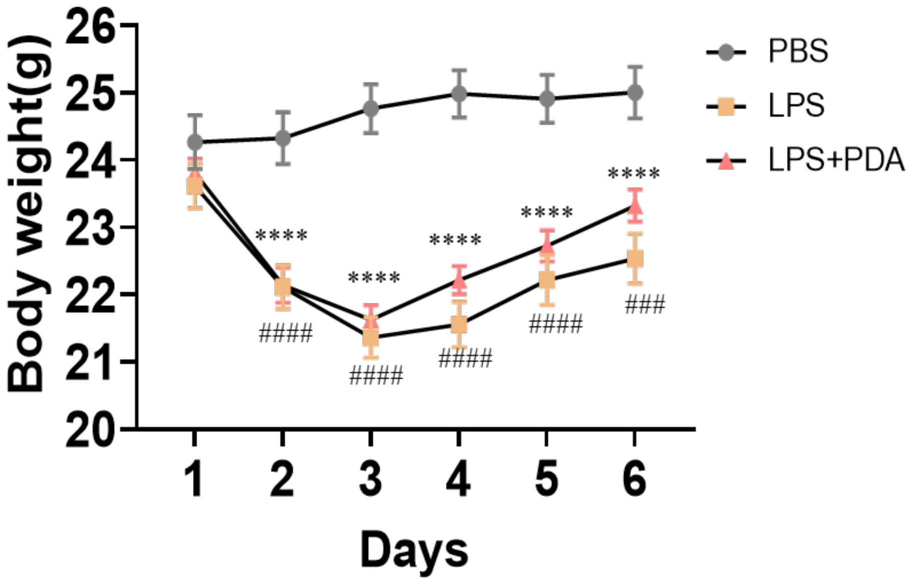

Figure. S11. The effects of LPS and PDA NPs in the body weight for mice. A graph showing the changes in body weight for PBS, LPS and LPS+PDA groups following their respective injections. The number of mice for each group is 12. PBS vs LPS: ****P<0.0001; PBS vs LPS+PDA: $^{####}$P<0.0001, $^{###}$P<0.001.

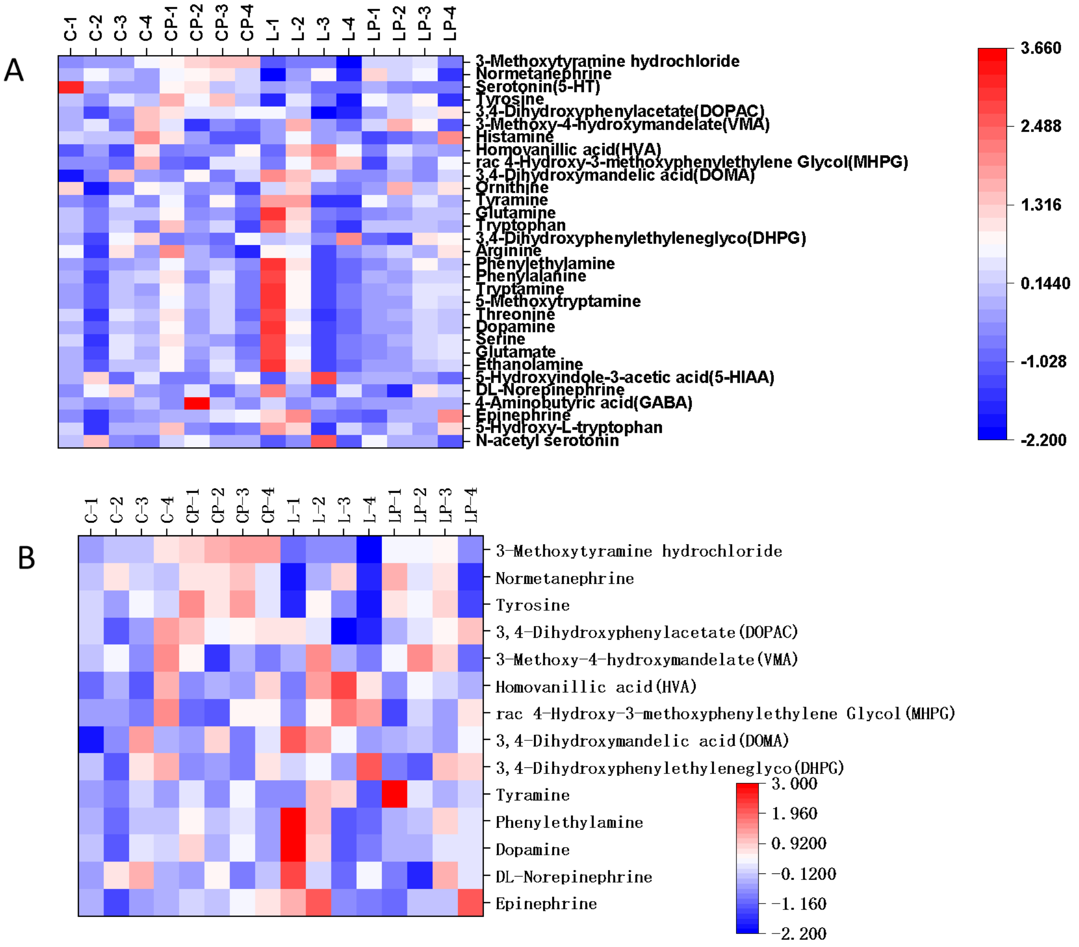

Figure. S12. Metabolites related with catecholamines in the hippocampus. (A): A heatmap depicting the levels of neurotransmitter-related metabolites in the hippocampus of mice on day 10. (B): A heatmap illustrating the levels of catecholamine neurotransmitter-related metabolites in the hippocampus of mice on day 10. (C1-C4: Control group, CP1-CP4: Control + PDA group, L1-L4: LPS + PBS group, LP1-LP4: LPS + PDA group).

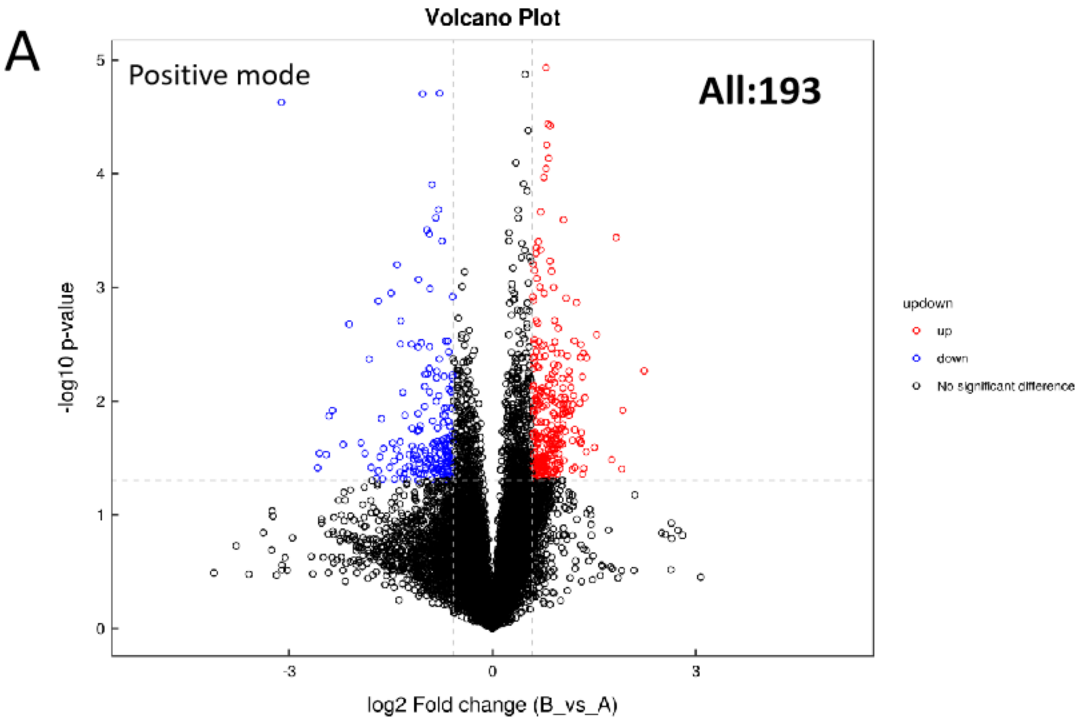

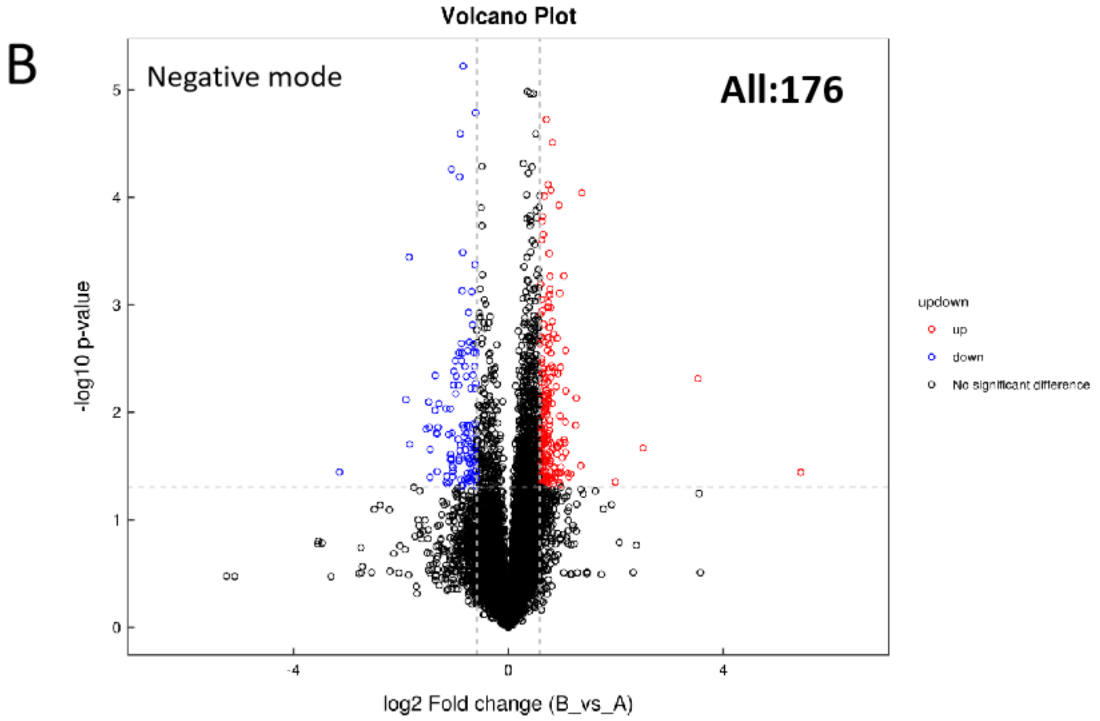

Figure. S13. Metabolites as indicated in the hippocampus of mice treated with PDA NPs for 9 days in positive and negative mode. The number of samples for each group is 8.

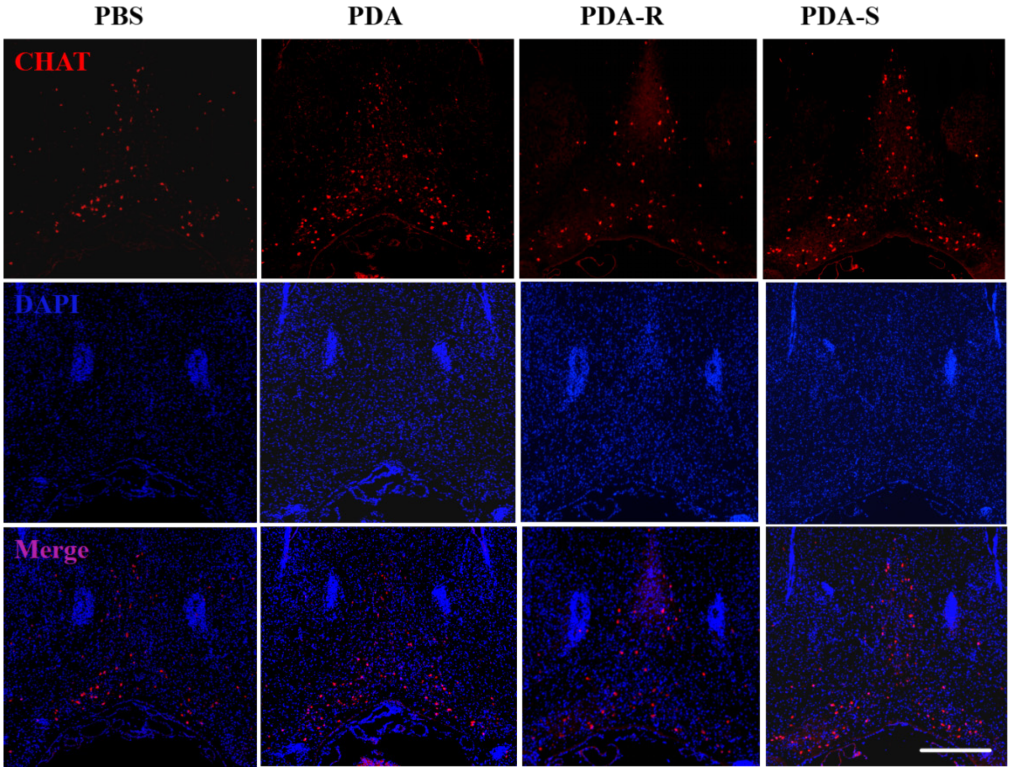

Figure. S14. Impact of PDA NP administration combined with intra-MS DA receptor antagonist on ACh synthesis in control mice. Representative immunofluorescence images illustrate the number of ChAT-positive cells in the medial septum (MS) region. PDA-R: PDA + raclopride (D_2 antagonist). PDA + S: PDA + SCH23390 (D1 antagonist). Scale bar: 500 μm.

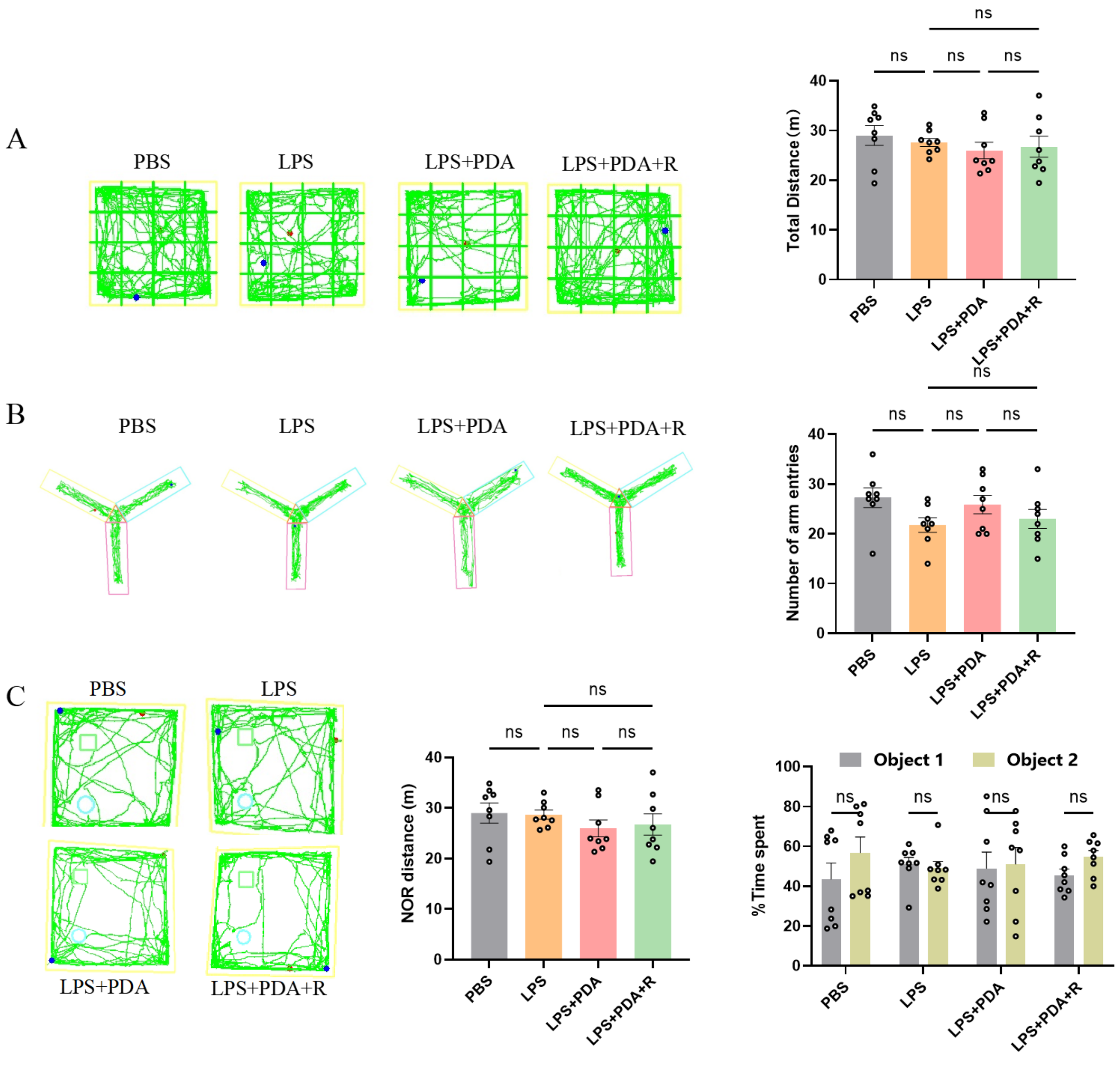

Figure. S15. Impact of combined PDA NP administration and intraperitoneal DA_2 receptor antagonist injection on ACh synthesis in LPS-treated mice. (A): Representative activity tracking from the open field test (OFT) with corresponding bar graphs (with individual data points) showing the total distance traveled during the 10-minute test. (B): Representative activity tracking in the Y-maze, along with bar graphs displaying the number of arm entries. (C): Representative activity tracking in the novel object recognition (NOR) test with corresponding bar graphs (with individual data points) showing the total distance traveled during the 10-minute test and the time ratio spent exploring object 1 versus object 2. (n = 8)

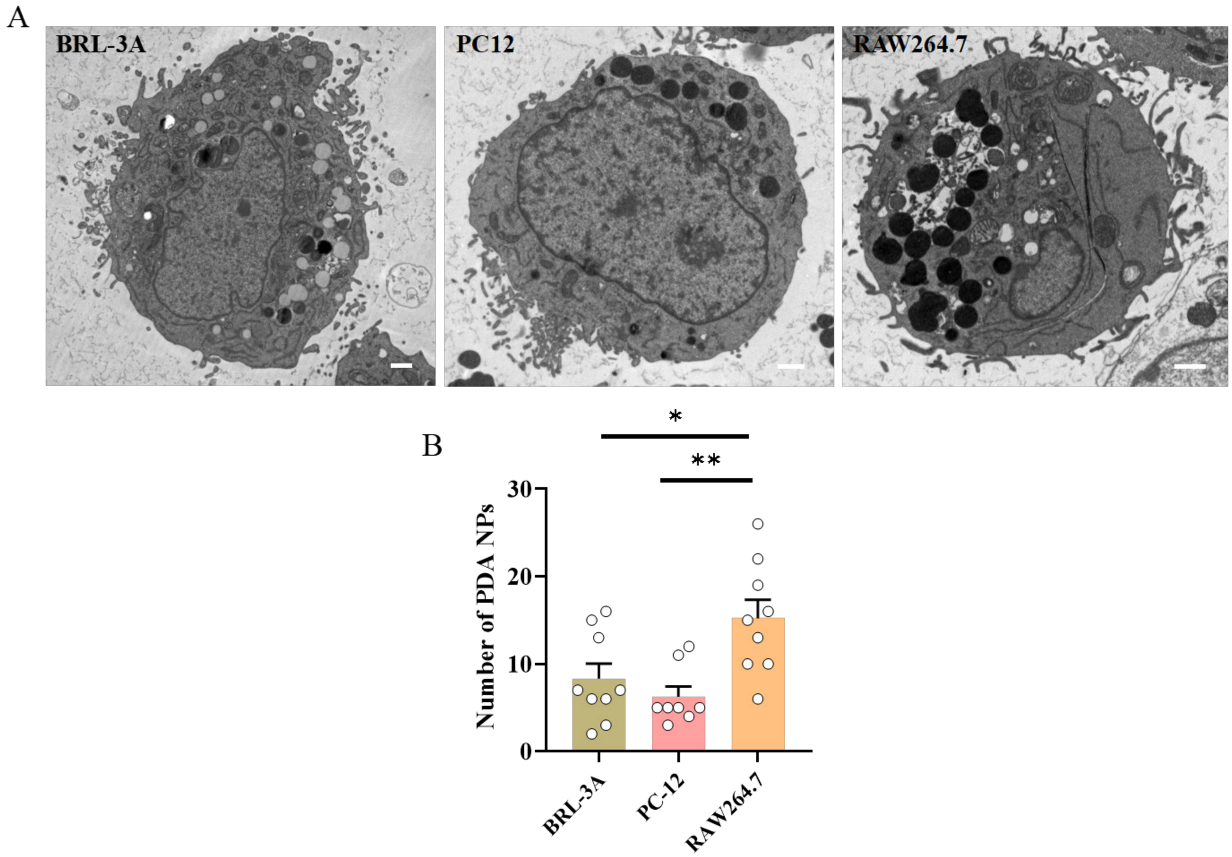

Figure. S16. (A): Representative TEM images of PDA NPs after 12 hours of co-culture with BRL-3A, PC12 cells, and RAW264.7 cells. Scale bars in the three imges were all 1 μm. (B): Corresponding bar graph showing the number of PDA NPs taken up by the three types of cells.*$P < 0.05$, **$P < 0.01$, n=9.

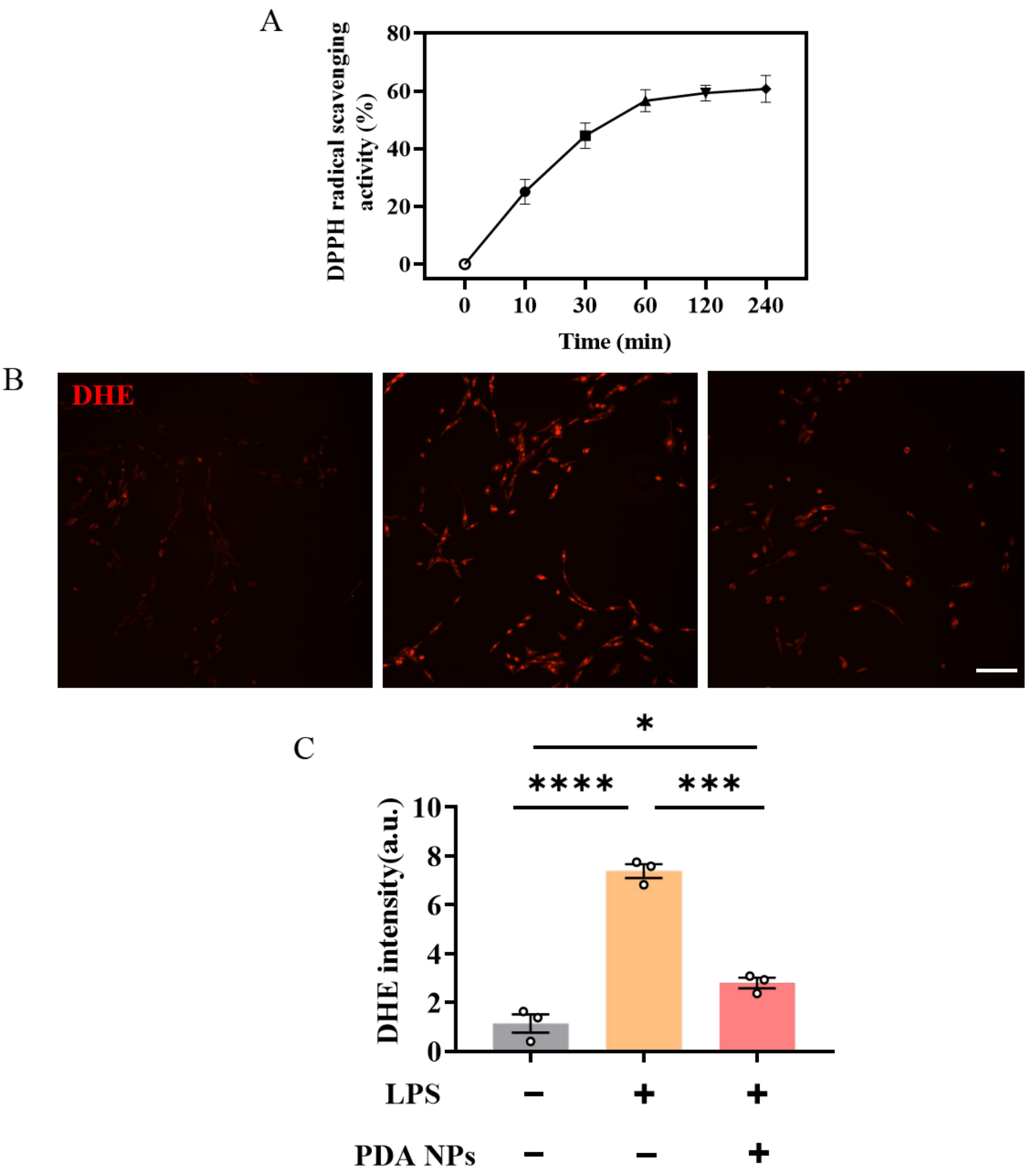

Figure. S17. (A) : Curved graph showing the DPPH radical scavenging activity of PDA NPs at defferent timepoint. (B) Representative immunofluorescence images (scale bar=200 μm) and (C) the corresponding bar graphs with dots showing ROS levels in the LPS-treated cells in the absence or presence of PDA NPs using DHE as the ROS probe. Data are presented as the means ± SEM. ****$P<0.0001$, ***$P<0.001$, *$P<0.05$. n=3.

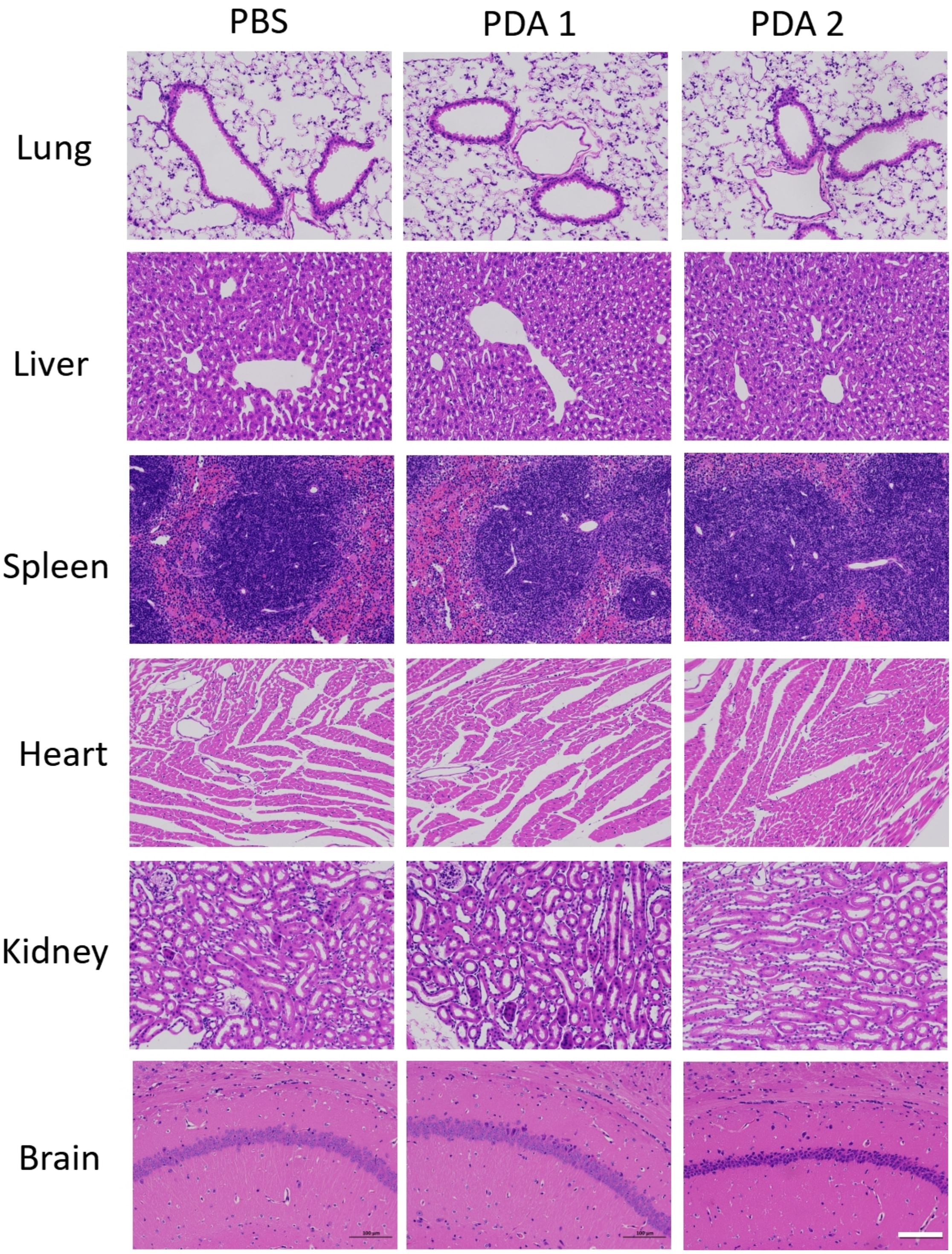

Figure. S18. Representative histological images of main organs following PDA NPs or PBS nine days post administration. Control mice were i.p. injected with PBS, or PDA NPs (5 and 10 mg/kg/day) for consecutive three days. Scale bar=100 μm.

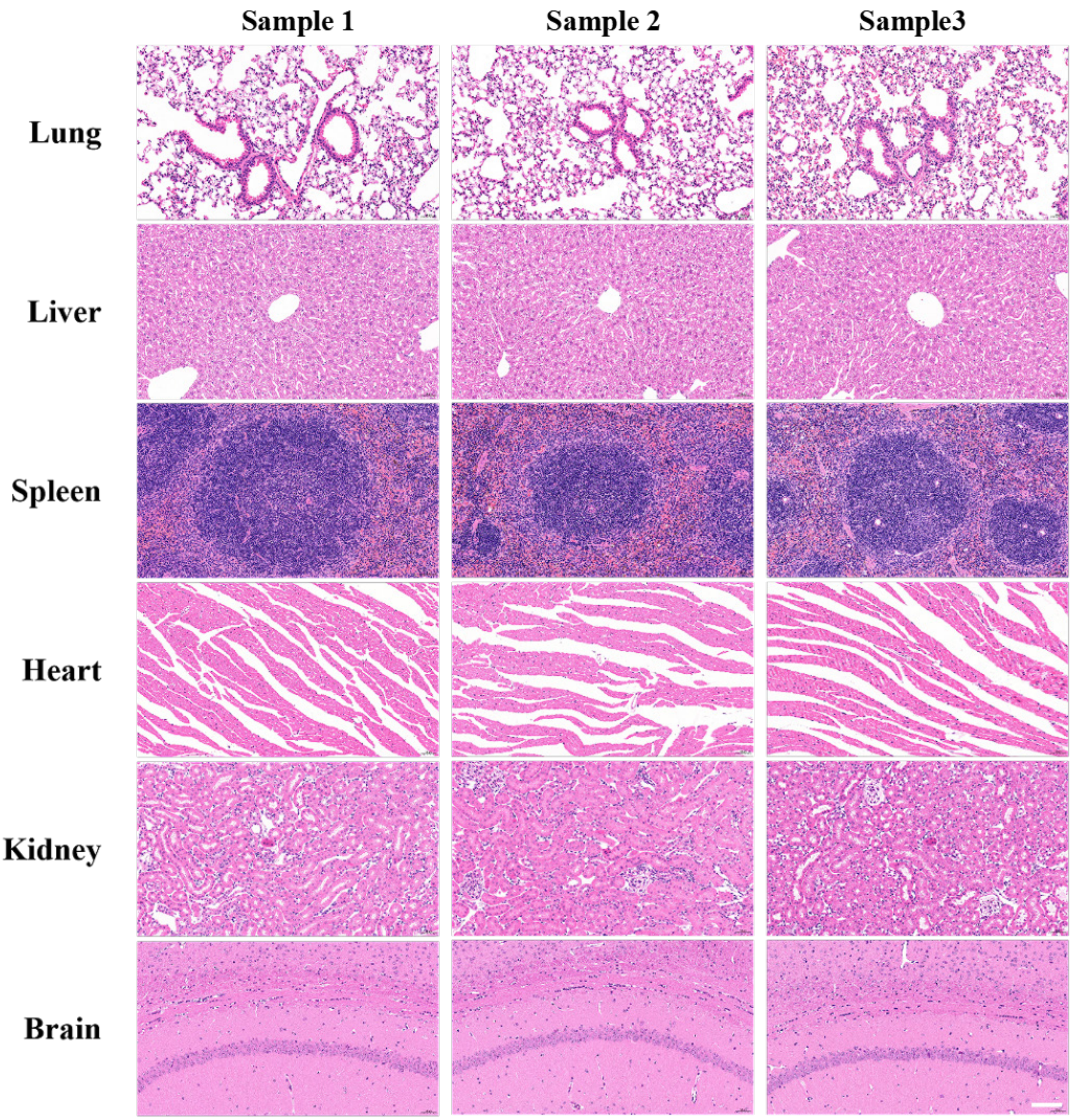

Figure. S19. Representative histological images of main organs following PDA NPs or PBS 60 days post i.p. administration. Mice were i.p. injected with PDA NPs (10 mg/kg/day) for consecutive three days. Scale bar=100 μm.

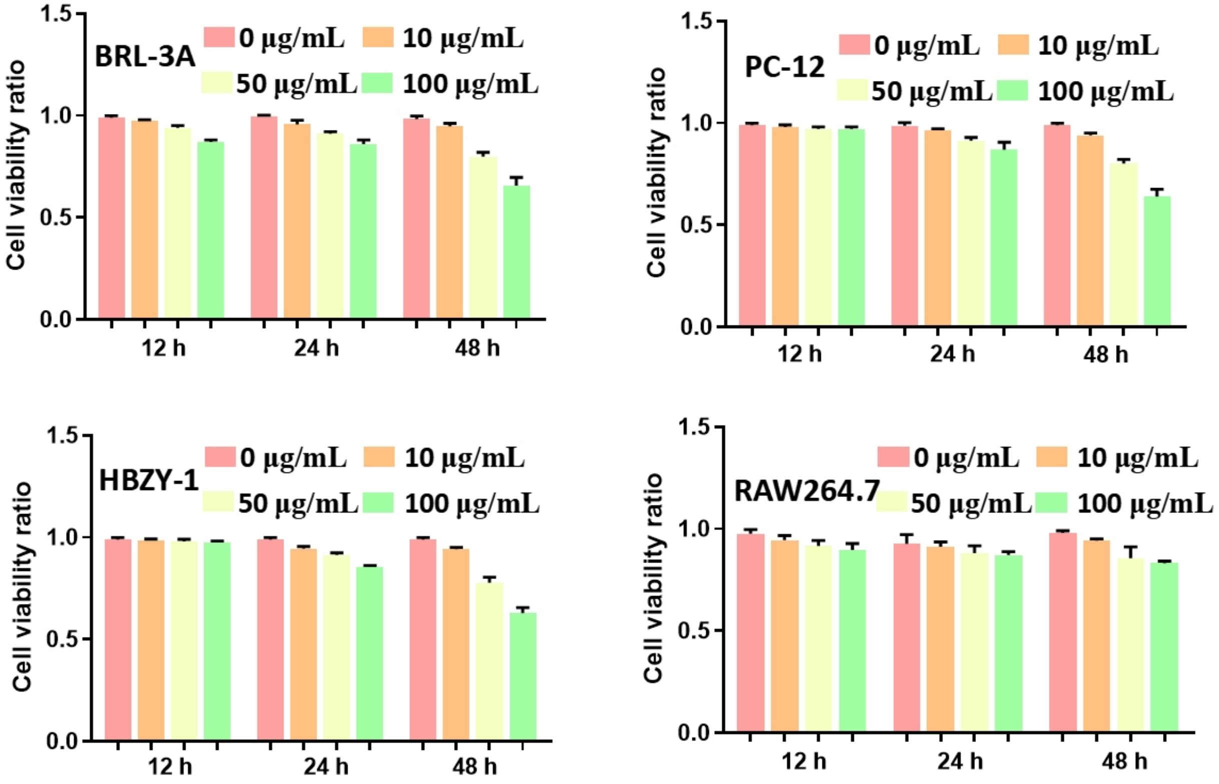

Figure. S20. Cell viability ratios of cells co-cultured with PDA NPs. Cell viability ratios of BRL-3A, PC-12, HBZY-1 and RAW264.7 cells co-cultured with PDA NPs at concentrations of 0, 10, 50, and 100 μg/mL, measured at 12, 24, and 48 hours. More than 0.8 of cell viability ratios was regarded as biocompatible. Data are presented as the mean ± SEM (n = 3).
